# Single-molecule capture, release, and dynamical manipulation via reversible electrokinetic confinement (RECON)

**DOI:** 10.1126/sciadv.adv8863

**Published:** 2025-09-17

**Authors:** Matheus A. S. Pessôa, Piotr Jakuc, Carolina Martins e Queiroz, Naomi Duggan, Ruiyao Liu, Seraphine Kautz, Sarah Ameur, Zezhou Liu, Wangwei Dong, Preethi Ravikumar, Sajad Shiekh, Han Cao, Michael Austin, Sara Mahshid, Deborah Fygenson, Walter Reisner

**Affiliations:** ^1^ Department of Physics, McGill University, Ernest Rutherford Building, 3600, Montréal, Québec, Canada.; ^2^ Department of Physics, University of California, Santa Barbara, CA 93106, USA.; ^3^ Dimension Genomics, San Diego, CA, USA.; ^4^ Department of Bioengineering, McGill University, Montréal, Québec, Canada.; ^5^ Department of Biomolecular Science and Engineering, University of California, Santa Barbara, CA 93106, USA.

## Abstract

We present a nanofluidic device enabling single-molecule confinement through free-energy landscapes created by dynamic electrical gating of embedded nanoelectrodes. Unlike static geometric confinement, this system uses a parallel electrode configuration with nanoelectrodes placed in a dielectric layer. Localized electrokinetic fields at electrode wells form tunable attractive potential wells for bimolecular capture. By modulating the voltage bias waveform, the device allows precise control over confinement dynamics, enabling molecular capture, release, and exposure to periodic or stochastic confinement regimes. This flexibility facilitates the study of biomolecular behavior under dynamically adjustable conditions, including controlled confinement fluctuations. The device can manipulate diverse analytes such as double-stranded DNA, liposomes, and DNA nanotubes and facilitates introducing molecules into confined environments intact from bulk while providing enhanced tunability. With the ability to implement tailored confinement profiles, this platform represents a versatile tool for probing molecular confinement and behavior in complex, dynamically varying environments.

## Teaser

The next generation of nanofluidic devices allows real-time spatial control of molecules.

## INTRODUCTION

Nanofluidic devices have emerged as a powerful tool in biomedical analysis, enabling highly parallelized and high-throughput single-molecule manipulation and characterization (*1*–*3*). Nanofluidic devices function by imposing steric confinement via pre-etched structures that geometrically restrict single-molecule position and conformation to lower dimensional spaces [e.g., two-dimensional (2D) slits (*4*), 1D nanochannels (*5*, *6*) and 0D cavities (*7*)]. Confinement can be harnessed to enable prolonged single-molecule interrogation [e.g., via local trapping that holds the molecule within the microscope field of view; (*8*)], drive enhanced binding kinetics [e.g., using small spaces to increase effective analyte concentrations and lower reactant mixing entropy; (*9*–*11*)], and perform conformational manipulations such as macromolecular stretching [e.g., double-stranded DNA (dsDNA) extension for genome mapping; (*3*, *4*, *12*, *13*)]. Moreover, confinement can be spatially modulated using nanofabrication techniques to vary etch depth (*14*, *15*); this induces variation in molecule configurational freedom (entropy) (*14*, *16*), giving rise to artificial free-energy landscapes possessing defined wells (traps) and hills (barriers). Polymer partitioning in such landscapes can be used to access single-polymer properties such as the molecule effective width (*17*). Quantifying thermally assisted escape of molecules and nanosized entities out of nanofluidic free-energy wells (*18*, *19*) at ultralow salt provides access to entity charge and size [with low salt conditions conferring sensitivity to screened electrostatic interactions; (*20*)]. Nanofluidic free-energy landscapes can also be used in nonequilibrium context to control molecular transport induced by constant electrical or hydrodynamic driving forces (*21*–*25*), for example, leading to size (*21*), topological (*22*), or charge-based separation (*26*).

Classic nanofluidic devices are intrinsically challenged by a static construction imposed by the overall etching/lithography protocols that fix confinement dimensions. This static “built-in” construction limits the ability to tune/modulate confinement in situ critical for optimizing device performance and achieving real-time control of confined molecules. In addition, the need to introduce molecules from bulk into static nanoconfined environments can lead to molecule fragmentation (*27*) and necessitate concentration steps to avoid lowered throughput due to the reduced number of molecules present in the reduced volume (*28*, *29*). Lastly, static construction prevents exploration of novel functionalities that might arise from the ability to perform confinement modulation on timescales comparable to molecular diffusion and relaxation times.

Active confinement approaches seek to overcome these challenges by introducing mechanisms for modulating confinement during device operation. In one approach, a piezo-controlled lens pusher (*8*, *27*) or pneumatic pressure (*7*, *30*–*33*) is used to deflect a thin bonding lid to drive molecules into etched nanoscale corrugations such as nanogrooves and nanopits. This approach can be used to adjust nanofluidic free-energy landscapes in situ by varying the degree of vertical confinement (slit-gap) between traps. The varying vertical confinement can modulate the escape time of nanoscale entities out of traps (*34*) and enable retention of trapped entities during cycled buffer exchanges (*29*) (i.e., ensuring that the surrounding slit is sufficiently thin to retain the trapped entity but sufficiently wide to pass necessary processing reagents). A related approach involves the use of deformable channel geometries based on elastomeric materials, enabling tunable confinement through mechanical actuation of the nanochannel walls (*35*). While lid deflection and nanochannel actuation-based confinement are powerful strategies and widely applicable, the mechanical nature of the operation poses serious fundamental drawbacks. On one hand, implementation tends to be cumbersome, either requiring specialized setups to control the lens pusher or complex fabrication approaches to mount free-standing thin films and/or fabricate elastomeric nanochannels. On the other hand, the mechanical nature of the deflection process limits the modulation response times, the lid deflection cannot be easily localized (i.e., it tends to simultaneously affect entire parallel arrays of trapping features), and lid deflection inevitably creates bulk hydrodynamic flows to displace the depressed volume away from the confined region.

An attractive possibility is to replace static built-in free-energy landscapes arising from modulated geometrical confinement with free-energy landscapes determined in part by spatially varying electric fields induced via external biasing (*36*). Electric fields exert forces on molecules in solution via a combination of electrokinetic effects (*37*) (electrophoresis and electroosmosis) and dielectrophoresis (DEP; arising from interaction of molecule-induced dipole with applied field) (*38*). With molecular response to applied fields occurring on subnanosecond timescales (*37*), use of external biasing would then ensure rapid landscape time modulation with customized dynamics supplied by inputting a user determined waveform. However, so far, obstacles arising from fundamental electrolyte solution physics and material limitations challenge nanofluidic exploitation of electric field–derived free-energy landscapes. A key fundamental challenge is that electric fields are screened in electrolyte solution due to formation of electric double layers at charged surfaces (*39*). This implies that maintaining extended electric fields at above ultralow ionic strength conditions (>~1 mM, where the Debye screening length $\lambda_{D}<10$ nm) requires current flows that can induce excessive electrolysis (*40*, *41*), leading to unstable device operation, gas evolution, and alternations in buffer chemistry. Insulating dielectric layers can control electrolysis (*42*, *43*) but are challenged in turn by the limited voltage they can withstand before breakdown [~1 V/nm in best case such as 500-nm-thick thermal oxide and typically <0.1 V/nm, e.g., for 20- to 30-nm atomic layer deposition (ALD) films; (*44*)]. For approaches incorporating local electrode gates, these challenges have in practice limited nanofluidic exploitation of electric field–derived free-energy modulation to ultralow ionic strength conditions (<1 mM). Thick insulating layers are necessary to avoid dielectric breakdown (*45*) necessitating high voltage (~100 V) such as in a recent study by Nicollier *et al.* (*36*) (using total insulation thickness of 0.72 μm with a reported Debye length of 13.3 nm). Operation modes that include dc/low frequency driving at >~1 mM ionic strength typically require designs where electrodes are placed away from active device regions, increasing device complexity and limiting the flexibility with which landscapes can be designed due to the need to ensure current flow into and out of the active regions. In one version of this approach, nanoanalytes are electrokinetically trapped at the inlet of a small aperture or pore that geometrically restricts the analytes’ passage [e.g., trapping at the inlet of 50-nm-diameter vertically aligned nanochannels bridging two perpendicular intersecting microchannels (*46*) or trapping of dsDNA at a nanopore embedded in a zero-mode waveguide for low input sequencing (*47*)]. In a second version, electrokinetic forces supplied by a quadrant electrode array are adjusted with feedback control to counteract Brownian motion (*48*, *49*). This method allows for spatial control of individual molecules without physical contact, making it suitable for studying molecular interactions and dynamics in real-time (*48*, *49*). However, this type of trap cannot be trivially parallelized into an array format, so it is unsuitable for generation of more complex landscapes. Lastly, although considerable research has been invested in alternative approaches based on DEP that can use pure ac driving (*38*), DEP suffers fundamentally from its unfavorable scaling with particle size [the DEP response depends on the third power of particle radius; (*38*)], often necessitating use of high applied voltage to drive DEP effects (*50*).

**Fig. 1.**
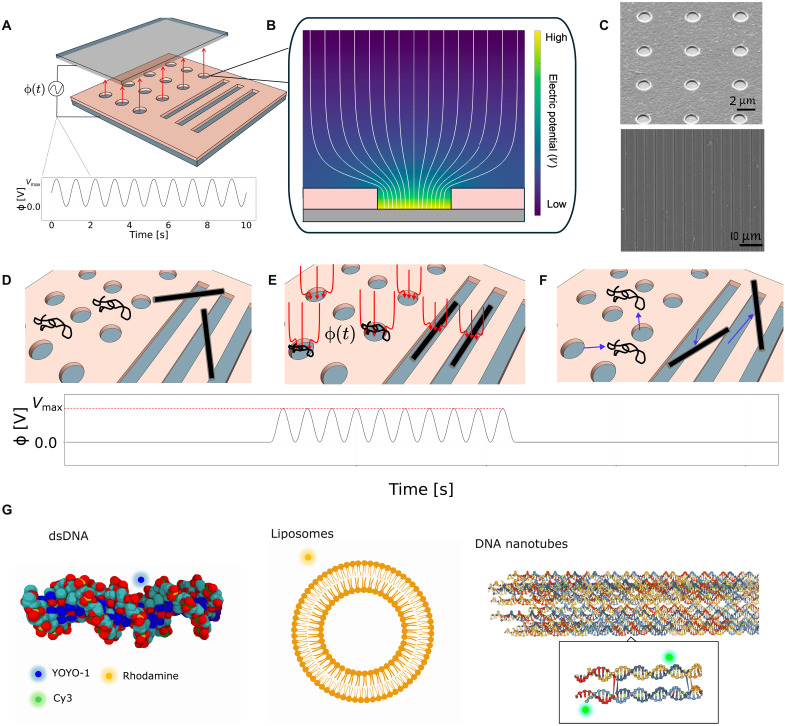
Reversible electrokinetic confinement device. (**A**) Schematic of electrokinetic device, indicating top lid electrode and nanowell features (both cavities and grooves) etched through dielectric layer that coats the electrode on the bottom device surface. The electrodes are connected to an external voltage generator that supplies an ACfp signal. (**B**) The magnitude of the electrical potential and example electric field lines in the vicinity of a well, showing the electric field funnel formed at the well upon biasing. (**C**) Scanning electron microscopy micrographs of cavity and groove structures. (**D**) When no field is applied, the nanoentities diffuse in bulk solution above the wells; (**E**) upon application of an electric field, they are attracted to the wells and are (**F**) released upon zeroing the field. (**G**) We demonstrate the approach with dsDNA, liposomes, and self-assembled DNA nanotubes. The dsDNA and liposome illustrations were made using BioRender, and the nanotubes illustrations were made using OxViewer (*59*).

Here, we demonstrate that it is possible to address key challenges involved in implementing time-modulated electric field–based confinement via dynamic gating of nanoscale electrode arrays. Our approach, which we term “electrokinetic confinement,” is based on a parallel plate construction with an upper uniform conducting surface vertically positioned over a lower surface containing arrays of nanoelectrode wells (Fig. 1A) (*51*). The wells are created by etching nanoscale openings in a dielectric layer [silicon nitride (SiN*_x_*)] coating an underlying conductive layer [indium tin oxide (ITO); Fig. 1] with passivation supplied by a nanometric ALD layer. A fluid-filled microfluidic flow cell is placed between the upper and lower surfaces and determines their vertical gap. Upon applying a voltage bias to the lower electrode with the upper electrode grounded, an electric field is induced in the gap (Fig. 1B), with electric field lines concentrated within and proximal to the electrode wells, giving rise to an electric field funnel at the well. Charged molecules and nanoentities of the sign opposite to that of the lower electrode biasing are then driven toward the electrode wells and remain confined in the well vicinity (Fig. 1, D to F). Critically, we find that use of an alternating fixed polarity (ACfp) bias signal leads to stable low-voltage electrokinetic operation without breakdown and gas evolution. The field confinement can be toggled on/off via simple on/off switching of ACfp biasing, leading to the ability to perform reversible capture/release of single molecules and nanoscale entities at the wells (Fig. 1, D to F), which we demonstrate with dsDNA constructs, self-assembled DNA nanotubes (*52*), and liposome vesicles (Fig. 1G). The electrode wells can be modulated in situ by adjusting signal voltage that is applied to all wells simultaneously. Taking advantage of the ability to apply customized dynamic biasing signals to our electric field confinement device, we show that low-frequency time modulation of field confinement can be accomplished by encoding slowly varying signals on ACfp waveforms via amplitude modulation. Customized biasing additionally enables stochastic driving via application of synthetic noise signals, leading to switching between confined and surface diffusive behavior. In analogy to classic nanofluidic designs, molecule partitioning can be accomplished by confinement of a single molecule in multiple electrode wells leading to formation of extended states with a molecule linker connecting filled wells. These capabilities lead to real-time dynamic control of single-molecule behavior at ionic strengths compatible with standard biological assays (~10 to 100 mM).

## RESULTS

### Device construction

Electrokinetic confinement is distinct from classic nanofluidic approaches and nanofluidic approaches harnessing lid deflection in that it avoids the need for vertical nanoconfinement at any point in its construction or operation. The electrokinetic confinement device is built on a borosilicate substrate coated with 100 nm of ITO and 300 nm of SiN*_x_* [plasma-enhanced chemical vapor deposition (PECVD)]. A single lithographic and etching step is used to define nanofluidic well features in the SiN*_x_* layer with the etching step timed to ensure complete penetration of the nitride layer within the features; the ITO layer is then completely exposed within each well. These features have a lateral geometry determined by the lithographic patterning, including lattices of circular nanocavities with variable radii (*r*_c_ = 250, 300, 350, 400 nm) and nanogrooves with multiple widths (*w* = 300, 400, 500 nm). A final nanometric thick passivation layer (TiO_2_, 2 to 3 nm) is applied via an ALD tool. An ITO-coated coverslip is used to form an upper electrode and device lid. The ITO coverslip is separated from the lower surface by patterned tape with a thickness of *d* = 30 μm (Fig. 1A). The patterned tape defines a microfluidic flow cell for introducing analyte containing solution. The device is secured to a 3D-printed chuck and mounted in a fluorescence microscopy setup with light-emitting diode–based multiwavelength illumination. The device is then connected in series to a waveform generator via silver wires secured to the ITO to provide synthetic bias signals (see section S1 for more details on chuck and flow-cell construction).

### Electrokinetic reversible confinement in a single cavity

**Fig. 2.**
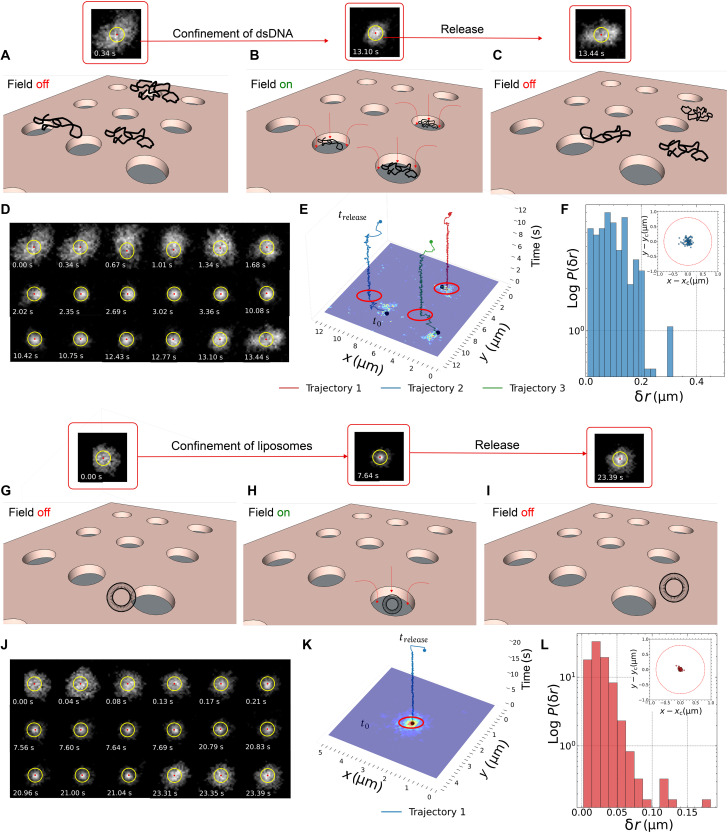
Single-cavity confinement of λ-DNA and liposomes. (**A** to **C**) DNA molecules, initially free in solution (A), are trapped in nanocavities under the influence of the ACfp signal (B) and then released by zeroing the field (C). (**D**) Time series of fluorescence micrographs of a λ-DNA molecule being trapped, held, and subsequently released from a nanocavity well (in yellow; diameter of 800 nm and depth of 400 nm), at 5 V, and with *f* = 100 kHz. The molecule dynamics can be quantified via FCM analysis; (**E**) molecule trajectory from initial frame (at time *t*_0_) until release (at time *t*_release_) is shown. (**F**) Histogram of the molecule displacement from the center of the trap δ*r*, with probability distribution of displacements *P*(δ*r*). This analysis gives insight into the degree of confinement of the molecule for a given bias signal. (**G** to **L**) Reversible confinement of liposomes in nanocavity wells of the same dimensions, including capture [(G) to (H)] and release (I) at 6 V and with *f* = 10 kHz. (J) Time series of fluorescence micrographs of a single liposome as it is captured, held, and released. (K) Corresponding liposome FCM trajectory and (L) resulting probability distribution.

We first demonstrate single-molecule capture and confinement in the electrokinetic wells with subsequent controlled release (see movie S1). Fluorescently labeled dsDNA molecules [λ-DNA, 48.5 kilo–base pair (kbp), stained with YOYO-1] are introduced into the flow cell using a syringe. Application of a positive ACfp bias signal at high frequency (100 kHz) to the electrode wells establishes field funnels at the wells and leads to DNA capture (Fig. 2, A, B, and D). The confinement is ceased when the ACfp signal is zeroed and the DNA is then released (Fig. 2, C and D). Critically, we find that signals with symmetric polarity (i.e., zero constant offset) do not lead to trapping. If DNA is captured via an ACfp signal and the signal is then toggled to a symmetric polarity signal, then the molecules diffuse from the wells. DEP is thus not the origin of the confinement effect observed here (*51*).

The consequent trajectory of λ-DNA during capture and release (Fig. 2D) can be visualized by plotting their fluorescence center of mass (FCM) as a function of time (Fig. 2E; for details on FCM calculation, see section S2). In Fig. 2E, three molecules are driven to separate wells, captured, and tightly confined. We define the center position of the well for *x* and *y* as the peak in the position distribution for all frames, and define δ*r* as the difference between the computed molecule FCM and the cavity center. Figure 2F gives the distribution of positions *P*(δ*r*); the inset shows the FCM distribution within the circular cavity.

**Fig. 3.**
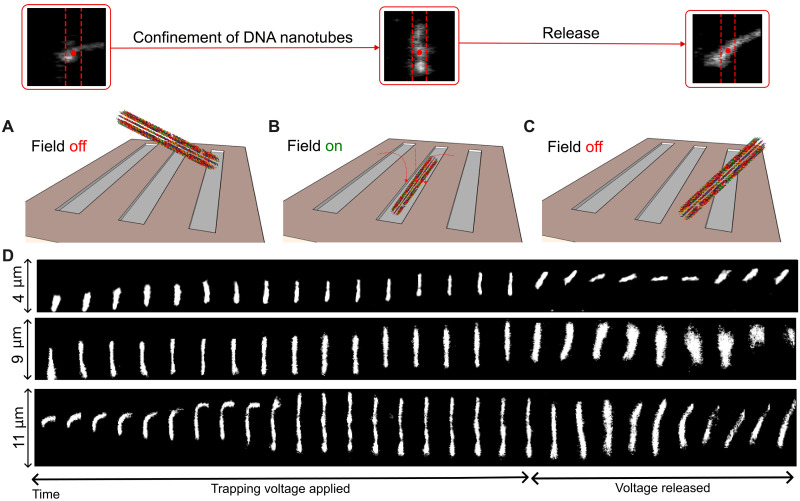
Reversible electrokinetic confinement of nanotubes. (**A** to **C**) Self-assembled DNA nanotubes, initially freely diffusing in solution (A), can be trapped in groove-like wells under the influence of the ACfp signal (B) and then released upon zeroing the field (C). (**D**) Time series of fluorescence micrographs showing capture, confinement, and release of nanotube structures in groove wells. Three different nanotube capture, confinement, and release events are shown. The grooves are 500 nm in width and 400 nm in depth, and trapping is performed using a sinusoidal ACfp signal with *f* = 100 kHz and *V*_max_ = 5 V.

Electrokinetic reversible confinement can be applied to manipulate a variety of nanostructures beyond dsDNA. For example, Fig. 2 (G to L) demonstrates the device principle applied to 100-nm-diameter liposome vesicles using circular cavities of size identical to the dsDNA example. The applied voltage in this case is higher than for λ-DNA, as the liposomes are smaller and more weakly charged. In addition, the trapping geometry can be modified to capture, confine, and release nanoentities with nonspherical geometries, such as self-assembled DNA nanotubes (Fig. 3). The nanotubes have a tubular shape with hollow core. The tube wall is composed of tiles consisting of a pair of dsDNAs that are held parallel to one another by a pair of “crossover” junctions separated by two full turns of the double helix along their lengths (*53*). To trap the nanotubes, we replace the circular wells with elongated groove wells (width *w* = 500 nm and depth *d* = 300 nm, as illustrated in Fig. 3, A to C). When no ACfp is present, the nanotubes diffuse freely in solution. When ACfp is applied, the nanotubes transit between an unconfined and confined state, involving either a global orientation of the nanotube to align along the groove axis, or a more complex dynamics, where the tube bends to allow a portion of the tube to align with the nanogroove, followed by a sliding motion of the tube along its length to ensure complete capture and alignment (Fig. 3D). Upon zeroing the ACfp signal, the tubes are released, as is evident by a reorientation of their axis away from the groove axis.

**Fig. 4.**
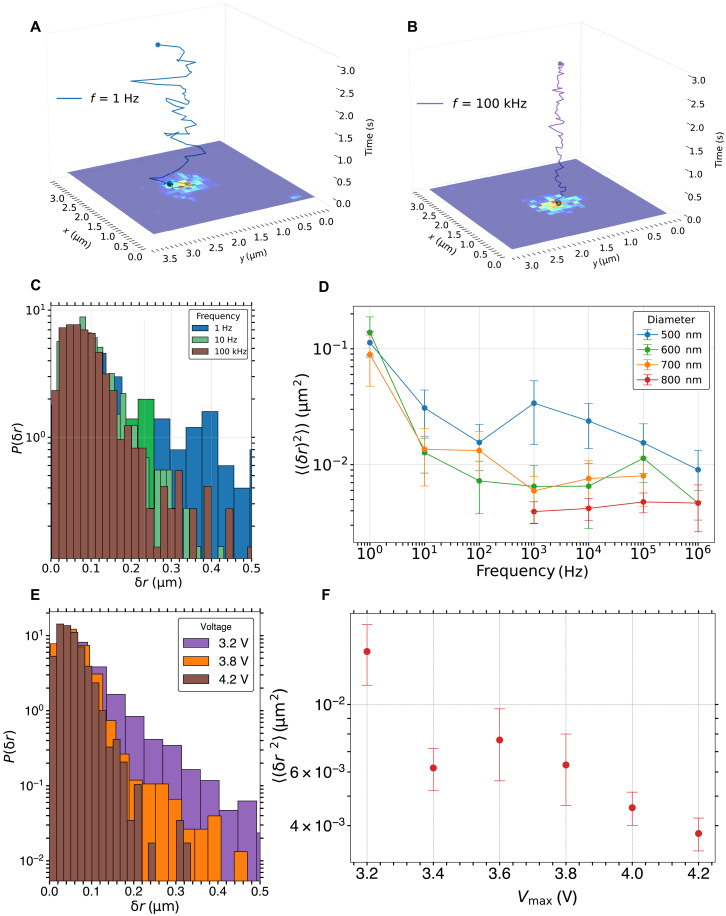
Frequency and voltage influence λ-DNA displacement. (**A** and **B**) The FCM trajectory of an electrokinetically confined λ-DNA at *f* = 1 Hz (A) and *f* = 100 kHz (B). The higher frequency suppresses periodic large excursions of the molecule from the well center, which arise during the longer low frequency cycles, leading to higher confinement. (**C**) The probability distribution of fluctuations from the trap center (δ*r*) for 1 Hz, 10 Hz, and 100 kHz and (**D**) the corresponding average mean square displacement $\langle {\left(\delta r\right)}^{2}\rangle$ for cavities with a fixed depth of 300 nm and diameters varying from 500 to 800 nm. The data points correspond to the quantity averaged over at least seven molecules; the reported uncertainties correspond to the standard error. (**E**) The *P*(δ*r*) for different voltages at fixed frequency (*f* = 100 kHz) and (**F**) the corresponding $\langle {\left(\delta r\right)}^{2}\rangle$ values.

The degree of confinement can be altered by varying the frequency of the ACfp signal (movie S2). Figure 4 (A and B) gives the time traces of the λ-DNA molecule FCM as a function of time for *f* = 1 Hz and *f* = 100 kHz. For low-frequency ACfp signals, the molecule can diffuse farther from the well center. As the frequency increases, the dynamics of confinement changes remarkably. At higher frequencies, the FCM trace is more stable, and the molecule does not diffuse as far away from the trap center. Integrating the fluctuation dynamics over at least 100 frames, we find the distribution of measured δ*r* values (Fig. 4C) and the corresponding variance [ $\langle {\left(\delta r\right)}^{2}\rangle$; Fig. 4D]. The variance as a function of frequency decreases and then plateaus over 10 to 100 Hz, indicating that achieved confinement saturates in this frequency range.

This frequency behavior can be rationalized by comparing the signal period *T* with the timescale for the polymer center of mass (CM) to diffuse out of the cavity ( $\tau_{d}$ ). The λ-DNA gyration radius $R_{g}\approx 700$ nm (*54*) is comparable to the cavity diameter of *d* = 800 nm. Thus, for voltages sufficiently strong to induce moderate compression, it is possible for the entire molecule to be pulled into and fit within the cavity; this strongly restricts the motion of the polymer CM. If $T<\;<\tau_{d}$ (i.e., working at high frequency), then the molecule will not have time to diffuse out of the cavity on the low voltage portion of the cycle. The molecule will remain in the cavity, and we expect minimum fluctuations of the polymer CM. However, as *T* approaches $\tau_{d}$ , then it will become increasingly probable on the low voltage portions of the cycle that the molecule CM diffuses vertically (i.e., in the *z* direction) out of the cavity into the portions of the electric field funnel above the cavity. This will lead to higher transverse fluctuations due to the relaxation of geometric constraints (i.e., the polymer will be less constrained in the *x*-*y* directions by the cavity sidewalls). The timescale $\tau_{d}$ can be estimated by finding the timescale for vertical diffusion over the cavity height $h:\tau_{d}≅h^{2}/D_{\mathrm{DNA}}$ where *h* is the cavity height and $D_{\text{DNA}}$ is the diffusion constant of the λ-DNA CM. Using $D_{\text{DNA}}\approx 0.5$ ${\mathrm{\mu m}}^{2}$ /s (*54*) and *h* = 300 nm, we find $\tau_{d}\approx 0.18$ s, leading to a critical frequency $f_{c}∼1/\tau_{d}=6$ Hz. Overall, this argument predicts a scenario that is consistent with what we observe in Fig. 4D: $\langle {\left(\delta r\right)}^{2}\rangle$ will be approximately constant down to frequencies within an order of magnitude of $f_{c}$ and then start to increase as the frequency approaches and drops below $f_{c}$ due to the increasing probability of molecule escape from the cavity.

The cavity size also plays a role in determining the behavior of the confined molecule. Somewhat counterintuitively, cavities with larger diameter give rise to a higher degree of confinement when compared to smaller diameters. This effect likely arises as the λ-DNA molecules cannot completely fit within the smaller cavities with diameter $<R_{g}=700$ nm, leading to a spillover into the field funnel positioned vertically above the cavity that increases the fluctuation in *x* and *y* relative to the cavity center. This conclusion is corroborated by an alternate analysis that quantifies the amount of “spillover” fluorescence present in the region between the cavity edge and the molecule’s furthest extent (see section S4). We find that the amount of spillover fluorescence decreases as the cavity width increases. The trend of decreasing CM fluctuations with increasing cavity width is also observed in Brownian dynamics simulations (*55*) of chains trapped in electrokinetic wells using a simplified model for the electric field (see section S7). We also explore the effect of varying signal voltage (Fig. 4, C and D); increasing signal voltage at a fixed frequency (*f* = 100 kHz) leads to an increase in confinement (Fig. 4, E and F).

**Fig. 5.**
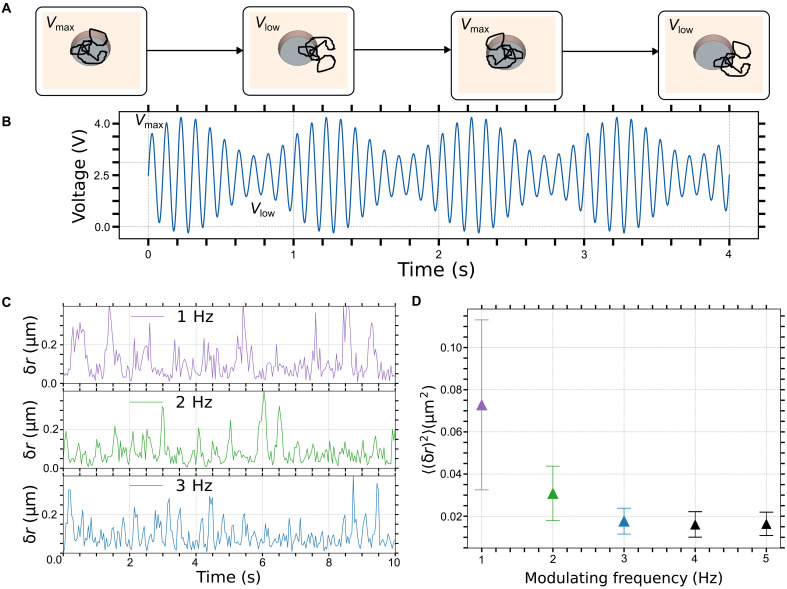
Confinement using amplitude-modulated signal. Concept of amplitude modulation demonstrated with cavity-confined DNA molecules, including (**A**) cartoon molecule/well system and (**B**) cartoon amplitude modulated voltage signal. Our signal has a base high frequency of *f* = 100 kHz, which we modulate sinusoidally between *V*_max_ = 4.0 V and *V*_low_ = 2.5 V at a modulating frequency *f_m_* = 1 Hz. (**C**) Time series showing deflection of λ-DNA from well center for 1, 2, and 3 Hz; the modulated signal leads to periodically weakening and strengthening confinement that induces periodic large molecule deflections. (**D**) The corresponding mean square displacement $\langle {\left(\delta r\right)}^{2}\rangle$ versus modulating frequency, demonstrating that confinement can be tuned by adjusting *f_m_*. Measurements at each frequency are based on an average of over 20 molecules. The cavity wells are 800 nm in diameter and 400 nm in depth.

Operating the device at higher frequencies (>1 kHz) avoids clear signatures of breakdown, leads to more stable and long-lasting device performance (up to 6 to 7 hours of continuous use), and ensures stable confinement without large periodic molecule excursions away from the well center. To ensure high-frequency confinement while retaining the ability to modulate confinement on physically relevant timescales (>10 ms, frequencies <100 Hz), we dynamically modulate the ACfp signal on longer timescales through amplitude modulation of the voltage waveform (Fig. 5, A and B, and movie S3):

$$\phi \left(t\right)=V_{0}+V_{\text{amp}}\left(t\right)\text{sin}\left(2\pi \text{ft}\right)$$ (1)

Here, $V_{0}$ indicates the offset required to achieve a fixed positive polarity signal, *f* is the underlying frequency of the ACfp signal, and $V_{\text{amp}}\left(t\right)$ gives the time-modulated amplitude. We choose a sinusoidal function for $V_{\text{amp}}\left(t\right):V_{\text{amp}}\left(t\right)=A_{0}\text{sin}\left(2\pi f_{m}t\right)$ with $f_{m}$ , a modulation frequency, so the well strength alternately becomes stronger and weaker (corresponding to deeper and shallower free-energy wells; Fig. 5B). Figure 5 (C and D) shows the resulting δ*r* for different modulation frequencies (Fig. 5C) and the resulting $\langle {\left(\delta r\right)}^{2}\rangle$ values (Fig. 5D). Figure 5 (C and D) indicates that faster amplitude modulation leads to reduced molecule excursion and higher confinement. This enables tuning of confinement in a way comparable to the use of nonmodulated sinusoids with varying frequency (Fig. 4D) but does so while maintaining a fixed voltage and underlying high-frequency ACfp driving signal.

### Electrokinetic reversible confinement in cavity arrays

**Fig. 6.**
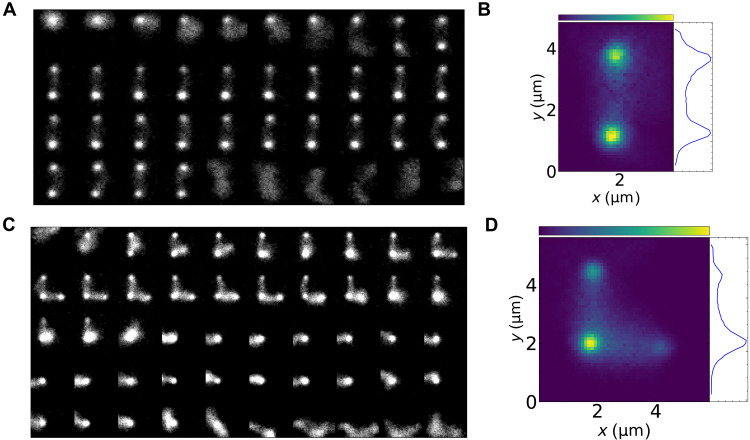
Molecular partitioning of T4 DNA molecules under electrokinetic confinement. (**A**) A time series montage of fluorescence micrographs showing formation of a dimer state and subsequent molecule release. The molecule is initially captured in a single well and then subsequently extends to the second well to form a dimer state. The molecule is released upon zeroing the biasing. (**B**) Image obtained by averaging fluorescence micrographs shown in (A). (**C**) In this case, the molecule transitions from a dimer to a trimer state that then decays to a single-well state prior to molecule release. (**D**) Image obtained by averaging fluorescence micrographs shown in (C). The well array had a 2-μm well spacing, 800-nm cavity diameter, cavity depth of 400 nm biased with an ACfp signal with *f* = 100 kHz and *V*_max_ = 4 V.

DNA molecules in complex nanofluidic environments featuring adjacent embedded cavities can reorganize their conformations so that contour is partitioned between multiple cavities. This leads to quantized conformations where a single polymer occupies multiple cavities with linkers connecting partially filled cavities (*14*, *17*). In our electrokinetic confinement device, analogous multiwell states can be formed for the given 2-μm cavity spacing with T4 DNA (166 kbp, 3.4× size of λ-DNA; see Fig. 6 and movie S4). In contrast to a classic nanofluidic device where the cavity structure is etched-in, in electrokinetic confinement, the state is controllable via the external bias signal, enabling capture of freely diffusing molecules into the multiwell state when the signal is switched on and release when the biasing is zeroed. Figure 6A gives an example of formation and release of a dimer well state. In this configuration, a single T4 DNA molecule occupies two adjacent cavities with a linker strand connecting the cavities; a relaxation process appears to occur after capture leading to a more uniform distribution of contour between the wells. Figure 6C shows formation of a trimer state (occupying three wells) that decays into a dimer state followed by a single-well state that is then released when the ACfp signal is zeroed.

**Fig. 7.**
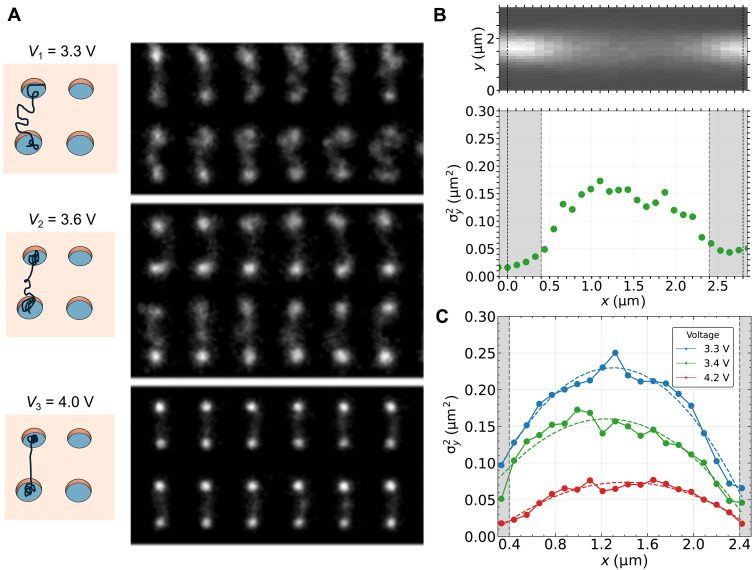
Quantifying fluctuations along DNA contour by varying applied voltage. (**A**) Time series of fluorescence micrographs showing the influence of voltage on T4 DNA dimer fluctuations between two adjacent cavities (spacing of 2 μm, diameter of 800 nm, and depth of 400 nm) for three different voltages (3.3, 3.6, and 4 V), with each frame being 0.042 s long. As voltage increases, the fluctuations decrease, indicating increased tension. (**B**) Top: Average fluorescence intensity between cavities, for 3.4 V. Bottom: The transverse fluctuation standard deviation $\sigma_{y}$ versus position along the linker. The dense dashed lines give the positions of the cavity centers, and the gray zones give spatial extent of the cavities. (**C**) The fluctuation variance plotted versus position along the linker with parabolic fits.

Electrokinetic confinement additionally allows for in situ fine-tuning of the multiwell state. For example, changing the ACfp voltage amplitude enables modulation of the well strength, which confers control over the tension in the linker (see movie S5). Figure 7A shows a dimer configuration for three different values of applied voltage ( $V_{1}=3.3$ V, $V_{2}=3.6$ V, and $V_{3}=4$ V). There is a clear suppression of transverse linker fluctuations and decreased fluctuations in the cavity intensities as the voltage increases. This effect arises as higher voltage increases the well trapping strength, pulling contour from the linker to the wells, which increases linker tension and suppresses chain fluctuations. The fluctuations are suppressed both in the transverse direction (the linker fluctuating back and forth perpendicular to the axis of extension) and longitudinally (contour transferred back and forth between the wells).

The linker tension can be quantified by measuring the transverse fluctuations of the extended DNA linker along the cavity separation axis (*56*). To do this, we subdivide the separation axis (*x* axis) into one-pixel windows (110 nm) and then compute the variance of the transverse fluctuations along *y* ( $\sigma_{y}^{2}$ ) for each window. The resulting fluctuations, compared to the average fluorescence intensity of the molecule spanning the two wells, are shown in Fig. 7B (dense dashed lines define the cavity centers; gray zones give spatial extent of the cavities). The fluctuations are suppressed within the well as a result of electrokinetic confinement and begin to increase toward the well edge. The transverse fluctuations then rise and peak at the midpoint between the wells; this corresponds to free transverse fluctuations of the linker strand joining the wells. Baba *et al.* have developed a model to describe the transverse fluctuations of an extended semiflexible polymer under uniform tension (*56*). In particular, they find that $\sigma_{y}$ depends quadratically on *x*, in agreement with what we observe (Fig. 7C). Moreover, they find that the maximum transverse fluctuation $\sigma_{y,\text{max}}$ follows an interpolation relation

$$\sigma_{y,\text{max}}={\mathrm{Ll}}_{p}\frac{F^{-1}}{4\left(1+\frac{3}{2}F^{-1}\right)}$$ (2)

with *F* as dimensionless stretching force $F={\mathrm{fl}}_{p}\;/k_{B}T$ . The quantity $l_{p}$ is the persistence length, *L* is the total contour of the chain under stretching force *f*, $k_{B}$ is Boltzmann’s constant, and *T* is the temperature. To obtain the force *f* from Eq. 2, we also need knowledge of *L*. To find *L*, we can introduce a second relation, noting that the stretching force *f* as a function of the chain extension *l* is described by the classic Marko-Siggia (*57*) interpolation equation

$$F=\frac{l}{L}+\frac{1}{4{\left(1-\frac{l}{L}\right)}^{2}}-\frac{1}{4}$$ (3)

The dimensionless force *F* can be eliminated between Eqs. 2 and 3, giving rise to an equation relating l/L and the dimensionless variance $\sigma_{y,\text{max}}^{2}/{\mathrm{ll}}_{p}$ , which we can invert to find *L* as a function of $\sigma_{y,\text{max}}$ , given knowledge of *l*. Equation 3 can then be used to find the corresponding force *f* that then determines the linker tension. The extension *l* corresponds to the extent of the linker region freely fluctuating under a uniform tension set by the electric field funnels at the cavity wells. Given that the fluctuations appear to abruptly rise at the edges of the wells (Fig. 7B), we choose to set *l* equal to the cavity-to-cavity spacing (2 μm); this assumes that the linker fluctuates freely immediately after leaving the well. This assumption may neglect subtle influence of the electric field funnel at the cavity edges; this effect could be captured by a molecular dynamics simulation directly incorporating a detailed model of the electric field at the well.

**Fig. 8.**
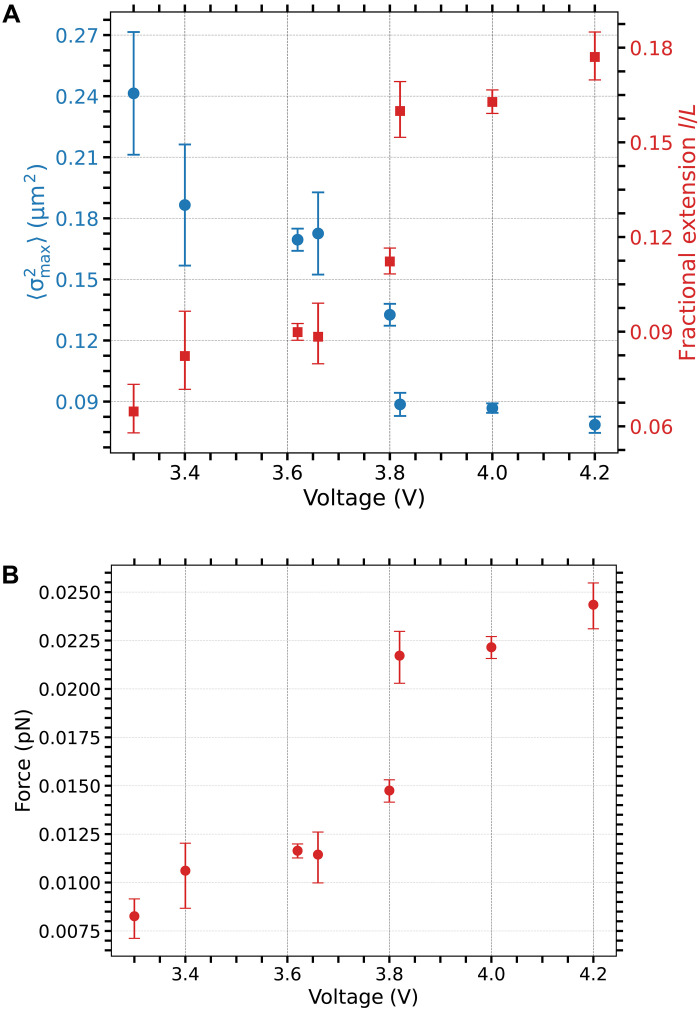
Fluctuation extension as a function of voltage and resulting applied force. (**A**) Blue circles show the maximum value of the transverse variance between the two cavity wells $\sigma_{y,\text{max}}^{2}$ as a function of $V_{\text{max}}$ . Red squares show the corresponding fractional extension l/L of the linker region under uniform tension. (**B**) Computed linker stretching force *f* as a function of voltage $V_{\text{max}}$ using the l/L values in (A). The number of molecules per voltage is *n* = 6 (3.30 V), *n* = 5 (3.40 V), *n* = 4 (3.62 V), *n* = 5 (3.66 V), *n* = 2 (3.80 V), *n* = 9 (3.82 V), *n* = 4 (4.00 V), and *n* = 7 (4.20 V). A decreasing $\langle \sigma_{\text{max}}^{2}\rangle$ is observed with increasing voltage, indicating higher force.

Figure 8A shows the obtained $\sigma_{y,\text{max}}^{2}$ values and corresponding l/L values; Fig. 8B shows the final computed values for stretching force versus voltage. Higher $V_{\text{max}}$ intuitively leads to a higher force with a force scale ~0.01 to 0.02 pN over the range of applied voltage. As, on average, the force exerted on the well by the extended linker must balance the force applied to the linker by the well due to the electrokinetic forces, the force-voltage curve equivalently gives the magnitude of the electrokinetic forces exerted on the DNA.

### Electrokinetic confinement with stochastic driving

**Fig. 9.**
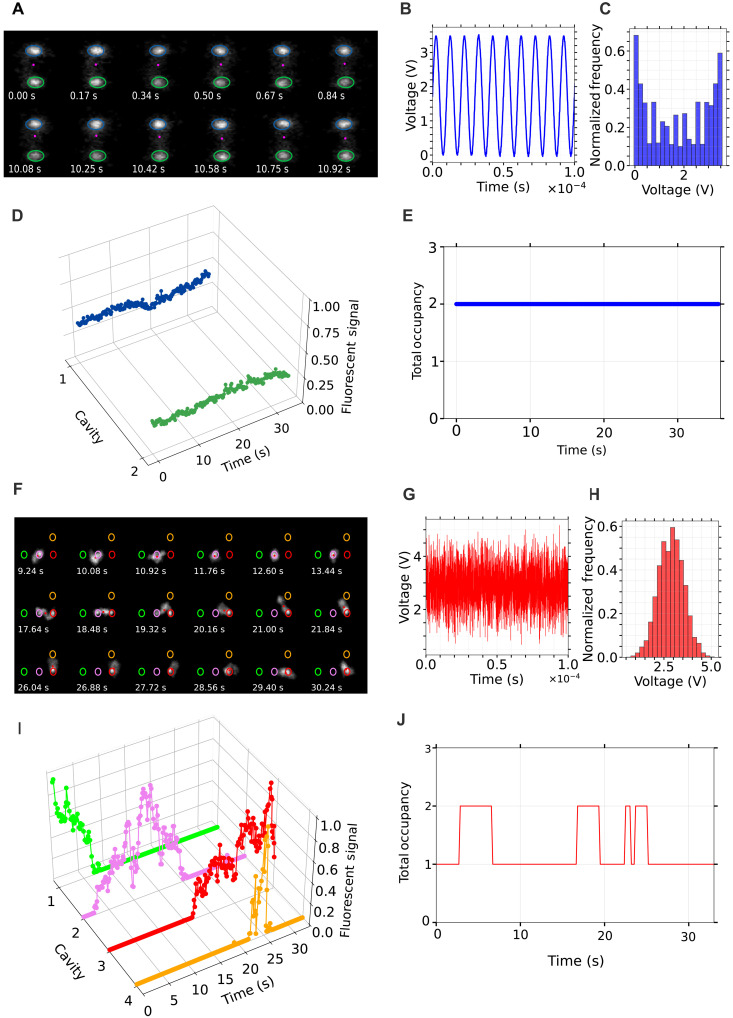
Stochastic driving influence on multiwell occupancy. (**A**) Time series montage of fluorescence micrographs, with stable confinement of T4 DNA molecules in the dimer conformation under the application of (**B** and **C**) an ACfp sinusoidal waveform (*f* = 100 kHz and $V_{\text{max}}=3.6$ V) for wells with diameter of 800 nm and depth of 400 nm. (**D**) The particular cavity wells occupied and (**E**) total occupancy (i.e., number of wells filled) remains stable across the experiment. (**F**) Dynamics under the influence of (**G** and **H**) Gaussian noise centered at $V_{\text{max}}$ = 2.9 V with a standard deviation of 0.66 V. (**I**) The molecules can jump between cavities giving rise to diffusive dynamics with (**J**) an occupancy that fluctuates in time.

Given complete freedom in choice of driving signal, one possibility is to operate the electrokinetic confinement devices with stochastic signals to simulate randomly fluctuating free-energy landscapes. While electrokinetically induced noise has been explored previously (*58*, *59*), in this past work, the noise modulation was applied to an external electric field to enhance DNA escape from a static two-cavity system formed using a classic nanofluidic approach (i.e., via pre-etched nanocavities in a nanoslit). In contrast, the electrokinetic confinement device enables direct stochastic modulation of the underlying free-energy landscape. As a demonstration of this capability, we investigate the effect of stochastic driving of a T4 DNA molecule in the free-energy landscape explored previously. In this landscape, upon driving with a constant-amplitude ACfp signal, the molecule adopts a two-cavity state (see Fig. 9, A to C) with a cavity occupancy that remains constant in time (see Fig. 9, D and E). In contrast, Fig. 9F shows the dynamics of T4 DNA molecule under electrokinetic confinement with a driving signal determined by Gaussian noise (Fig. 9, F to J, and movie S6). The Gaussian noise signal leads to molecule surface capture from bulk and is sufficient to hold the molecule close to the surface, but in this case, the dynamics is quite distinct with multiple transitions between cavities, captured by the fluorescent signal within each cavity as a function of time (Fig. 9F). The total occupancy as a function of time (Fig. 9I) varies between one- and two-cavity states, reflecting conformational transitions driven by the stochastic confinement modulation. For example, at *t* = 9.24 s, the T4 DNA occupies a single cavity (Fig. 9, F and I, magenta) and in later frames is partitioned into a second cavity and transits to a dimer state (red) then transitions back to a single cavity (red), briefly enters a second cavity (orange) to form a second dimer configuration and then retreats back to a single cavity. This constant jumping between one- and two-cavity states drives molecule transit through the lattice without forming stable long-lasting dimer conformations as observed for the constant-amplitude ACfp signal.

**Fig. 10.**
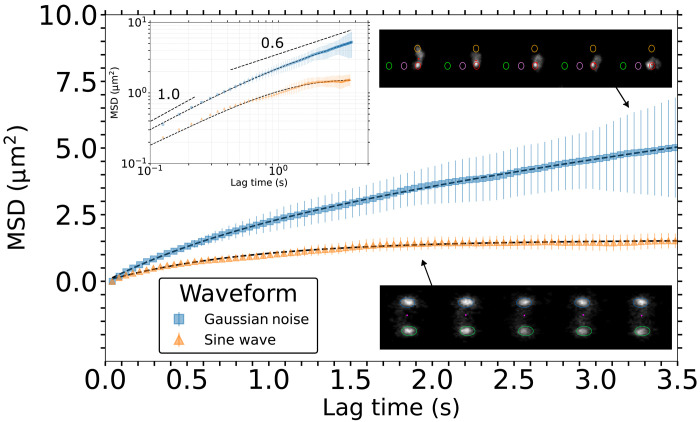
Influence of stochastic driving on MSD. The MSD for T4 molecules in the cavity well array under influence of sinusoidal ACfp (orange) and Gaussian noise driving (blue). The dashed lines show fits to the MSD models outlined in the text [ACfp results fitted to confined diffusion model (Eq. 4) and Gaussian noise results fitted to subdiffusive model (Eq. 5)]. The inset shows results on a log-log scale, clearly indicating normal diffusive behavior for low lag time for both driving signals, but a diverging behavior at high lag time, with ACfp driving giving rise to a constant MSD (consistent with long-time confinement) and noise driving giving rise to a power law MSD with exponent less than unity (indicating subdiffusive behavior). In both insets, the red and magenta circles correspond respectively to the FCM of the system for the Gaussian noise (top) and the sine wave (bottom).

We can use the mean square displacement (MSD) obtained from FCM tracking to more explicitly quantify the difference in dynamics between fixed-amplitude ACfp driving and driving via Gaussian noise (Fig. 10). Fixed-amplitude ACfp driving leads to long-time confinement (Fig. 9, B to E) and, hence, saturation of the MSD in the long-time limit, leading to an MSD curve that can be modeled via a classic MSD profile for confined diffusion

$$\;\text{MSD}\;\left(t_{\text{lag}}\right)=A\left[1-\text{exp}\left(-t_{\text{lag}}/\tau \right)\right]$$ (4)

where the quantity *A* is the maximum MSD relating to the confined dimensions of the well and τ is a timescale over which the molecule transitions from free to confined diffusion (i.e., begins to feel the effect of confinement). A best-fit gives τ=0.57±0.03 s. In the presence of Gaussian noise, the molecule is no longer confined at long times but instead exhibits long-term subdiffusive behavior. To model this subdiffusive MSD, we introduce a model that explicitly transitions between normal diffusion and subdiffusion with subdiffusive exponent δ

$$\begin{array}{c} \;\text{MSD}\;|\;\left(t_{\text{lag}}\right)=A{\left[1-\text{exp}\left(-t_{\text{lag}}/\tau \right)\right]}^{\left(1-\delta \right)}t_{\text{lag}}^{\delta } \end{array}$$ (5)

This leads to a δ=0.6195±0.0034 , indicating subdiffusive behavior ( δ<1 ), and τ=0.29±0.02  s. Subdiffusion is of fundamental interest in soft-matter physics and also biophysically important; subdiffusion in cells (*60*) is widely believed to arise from transient trapping of molecules in cytoplasmic cages formed as a consequence of molecular crowding (*61*, *62*) or within pores in the cytoskeletical network (*63*). In these examples, the confinement is not static but subject to temporal fluctuations [e.g., cages transiently forming/varying in size or cytoskeletal fluctuations induced by active processes such as motor dynamics (*62*)]. The ability to generate subdiffusive dynamics by modulating the electrokinetic confinement via a synthetic stochastic signal suggests that RECON could be used to model more broadly how confinement fluctuations lead to anomalous diffusion and precisely elucidate how variation in noise properties alters the dynamics.

## DISCUSSION

In summary, we have developed an electrokinetic confinement device that enables capture, confinement, and release of single molecules and other nanoscale entities such as vesicles and DNA nanotubes. In contrast to previous nanofluidic approaches based on static or variable steric confinement, electrokinetic confinement relies on dynamic electrical gating and can thus be fine-tuned in situ and rapidly modulated via application of external voltage signals. Simple on/off modulation leads to basic capture/release functionality, but potential control options are extremely large due to the ability to drive the device via synthetic voltage signals. For example, switching between periodic and stochastic driving can toggle the device between confined/localized and 2D subdiffusive regimes of motion. In addition, electrokinetic confinement does not require that nanoentities be introduced into highly confined environments for capture/manipulation, simplifying molecule loading, device fabrication, and device operation via the increased accessibility of the analytes. In the electrokinetic confinement device, molecules are introduced into a microscale flow cell and pulled vertically into the free-energy landscape with no need to adjust the flow-cell geometry. Lastly, the electrokinetic confinement approach shares with other nanofluidic approaches the ability to modify free-energy landscapes spatially by control over the electrode-well geometry and spacing (e.g., to achieve configurations where a molecule is partitioned between multiple wells), while simultaneously expanding the achievable control by enabling dynamic time modulation of these complex landscapes.

A key advantage of the electrokinetic confinement approach is that it enables low-voltage electrical modulation of free-energy landscapes in solutions with >1 mM ionic strength, critical for widespread application. As capture is only evident with fixed polarity driving, the origin of the effect is electrophoretic rather than dielectrophoretic. Preliminary explorations of the mechanism suggest that a time-averaged electric field is generated via electrochemical reactions that lead to a small but finite time-averaged current through each well (see section S5). This current can be understood in electrostatic terms as generating an electric field via fixing the electric field normal component at the well floor in terms of the well-surface current density divided by solution conductivity $\sigma_{c}$ (see section S6 for discussion of electric field within a cavity). In particular, we measure under ACfp driving a current that grows steeply above 2 V, consistent with water electrolysis, which is thermodynamically allowed at a minimum cell voltage of 1.23 V (sum of half cell potentials for water splitting) and typically emerges at voltages ~2 V due to the overpotential required to drive reaction kinetics and reactant transport (*64*). Use of higher-frequency (100 kHz) ACfp driving lowers the electrochemical current, providing a current sufficiently strong to drive capture at the wells but low enough to avoid gas evolution. Specifically, we measure a current density of ~1 pA/${\mathrm{\mu m}}^{2}$ at 100 kHz at voltage levels sufficient to drive molecule capture (see fig. S5B).

This current can be directly connected with the order of magnitude of the force scale observed in our measurement of linker tension in the two-well system. To estimate the electrokinetic force applied by the well, a classic argument by Adjari and colleagues (*65*) suggests that a polyelectrolyte experiencing electrokinetic forces under the influence of an external mechanical force $f_{\mathrm{ext}}$ satisfies $f_{\text{ext}}=\xi \left(v-\mu E\right)$ where ξ is the polymer friction factor, *v* is the net velocity, μ is the mobility, and *E* is the electric field. Let $f_{\mathrm{ext}}$ be the tension in the linker spanning the two wells. If there is an equilibrium between the field induced force and $f_{\text{ext}}$ , then *v* = 0 and $f_{\mathrm{ext}}=-\xi \mu E$ . Thus, the electrokinetic force applied by the well $f_{\text{EK}}=\xi \mu E$ . Following classic arguments used to elucidate polymer dynamics in confined systems like slits and nanochannels (*66*), we expect hydrodynamic interactions to be screened over a length scale on order of the well diameter *d*. Thus, ξ≈6πηd , yielding a net electrokinetic force $f_{\text{EK}}\approx 6\pi \eta d\mu E$ . The electric field is written as $E=J\;/\sigma_{c}$ where *J* is the measured current density (3.8 pA/${\mathrm{\mu m}}^{2}$ at 3.5 V; see fig. S5B) and $\sigma_{c}$ is the buffer conductivity (measured to be 0.768 mS/cm). The net electrokinetic force calculated using this argument, $f_{\text{EK}}\approx 0.03$ pN, is on order of the measured force (~0.01 pN; Fig. 8B), using η = 1 mPa·s, *d*_c_ = 800 nm, and DNA mobility $\mu =4.5\times 10^{-4}$ cm^2^/V [measured in tris-boric acid–EDTA buffer (TBE); (*67*)]. Note that this calculation is valid only in an order of magnitude sense; detailed simulations for our geometry incorporating realistic hydrodynamics are needed to obtain the correct numerical prefactor of the cavity-confined DNA friction factor ξ (6π is certainly an overestimate as it technically applies exactly only to hard spheres).

One question is the potential role of electroosmosis. While electrophoretic effects are described simply as the product of a mobility coefficient and an electric field (*37*), electroosmosis can create complex flows at junctions of features of varying dimensions/geometries. Electroosmosis is also coupled to the degree of local surface charging, which is itself a function of the local electric field (*68*). In a situation where the cavity surface charge and zeta potential are nonuniform, as is the case here, simulations suggest that complex vortex flows can be generated within the cavity (*69*). In our system, the electric field arising from the current flow through the well has a radial component at the well floor that can potentially generate inward-directed electroosmotic flow, given a positive charging of the well by the applied voltage (fig. S6D). As the device top and bottom surfaces are impermeable to liquid, this would necessarily create a local recirculating flow profile located at the well. This recirculating flow most likely does not contribute to molecular capture; closed circular streamlines could introduce analytes into the well from bulk solution, but they would then necessarily drive the analytes out on the upward trajectory, where there would be a substantial increase in the probability that the analytes could be lost to diffusion. However, once the analytes are captured and held vertically in the well via electrophoretic forces, electroosmotic flow could provide additional radially directed forces that would influence the distribution of the captured analytes. In particular, the observed distribution of captured liposomes with radial position in the well (Fig. 2L) is tighter than we would expect if electrophoresis was the only mechanism present. Electrophoresis acting alone would give rise to an almost uniform distribution of vesicles across the well as the radial field components are quite weak compared to the vertical field components, suggesting an additional possibly electroosmotic mechanism.

We believe that the intertwined electrical, chemical, and hydrodynamic effects that underlie our device performance could be addressed quantitatively via modeling strategies developed in the context of ac microfluidics (*70*–*75*). These approaches solve the coupled Poisson, Stokes, and Nernst-Planck equations for all ionic species (*71*–*75*), with boundary conditions imposed at the electrode interface that reflect the presence of capacitive charging and electrochemical charge transfer [e.g., modeled via Butler-Volmer (*72*–*75*)]. These studies find that nonequilibrium effects can influence the charge screening layer. The classic electric double layer (Stern or compact plus diffuse) can be extended by the presence of a frequency-dependent diffusion layer ( $\delta ∼\sqrt{D/\omega }$ ) that alters the electroosmotic flow profile (*74*). We obtain a δ of tens of nanometers using an ionic diffusion constant $D∼10^{-9}$ m^2^/s (*76*); this is less than the cavity height (300 to 400 nm) but does not constitute a truly infinitesimally thin layer relative to cavity dimensions. These considerations would leave our key qualitative conclusions in place but would necessitate full modeling for satisfactory quantitative understanding, for example, to elucidate key questions concerning the exact extent of the screening layer under our driving conditions; how the concentration of buffer species (tris and borate ions, “indifferent” electrolytes in electrochemical terms) affect this screening layer; and quantitative prediction of generated electroosmotic flows.

In the future, the electrokinetic confinement approach could be potentially applied to simplify and enhance a range of single-molecule/nanostructure manipulation and confinement applications. Long-time imaging of nanoentities can be performed via confinement in parallel electrokinetic well arrays, with the added capability of being able to assess perturbations in molecular dynamics induced via dynamic confinement modulation. The ability to release following capture could enable analyte sorting directed by assessment of measured molecular/structure properties. In particular, a natural next step is to fabricate devices in which the wells are individually addressable (e.g., which could be implemented by patterning the underlying ITO layer). With individually addressable wells, analytes captured in specific wells could be selectively retained and released. In addition, replacing electrolysis-based electrochemistry with a redox-active agent added to the buffer, an approach used in electrical loading of zero-mode waveguides, might achieve higher fields at lower voltages (*77*). DNA mapping applications may be enabled by stretching DNA between adjacent electrokinetic wells. Applications such as single vesicle analysis rely on chemical absorption of vesicles onto surfaces followed by repeated labeling/washing steps; the electrokinetic confinement approach could be used to perform cyclic and reversible absorption with higher surface binding density. From a soft-matter and biophysics point of view, this system may enable exploration of polymer dynamics in confined/fluctuating landscapes with landscape time modulation custom-controlled via design of synthetic driving signals.

## MATERIALS AND METHODS

### Device nanofabrication

Electrokinetic confinement devices were fabricated at the McGill Nanotools Facility and at the Institut National de la Recherche Scientifique. Borosilicate glass substrates coated with ~180 ± 10 nm of ITO (measured using ellipsometry) were cleaned via acetone and isopropyl alchohol. The devices were then coated with PECVD SiN*_x_* (with thickness of 314 ± 10 nm, measured via ellipsometry, with uncertainties arising from spatial variability of deposited material verified by reflectometry). The etched wells were then formed using a process based on electron beam lithography and reactive ion etching (CHF_3_, Ar, and O_2_ etching chemistry using a ZEP resist mask). Following etching of the wells, ALD was used to produce a passivating layer of 2 to 3 nm TiO_2_ using tetramethylammonium-based precursors at 250°C.

### DNA and liposome sample preparation

TBE buffer (1×) was mixed with λ-phage DNA (48.5 kbp) and T4 DNA (166 kbp) at a concentration of 10 μg/ml with the DNA YOYO-1 stained at a 1 to 10 dye per base pair ratio. The liposomes (commerciallly available from Formumax) consist of 1,2-dioleoyl-*sn*-glycero-3-phosphocholine and cholesterol; they were fluorescently labeled with lissamine rhodamine B 1,2-dihexadecanoyl-*sn*-glycerol-3-phosphoethanolamine dye triethylammonium salt [DOPC/CH-OL/Rh-DHPE at 54:45:1 mol/mol, with 50-mM lipid concentration and 0.5-mM (0.67 mg/ml) Rh-DHPE proportion]. The liposomes have a distribution of diameters of 100 to 120 nm.

### DNA nanotube sample preparation

The SEs core nanotubes are diluted in tris-acetate-EDTA (TAE) buffer (1×) (*78*). Prior to experiments, we incubated the surface of the nanofluidic chips in 30 μl of 50-mM sodium phosphate buffer for surface conditioning (*79*). Then, 2 μl of the SEs DNA nanotube solution was added to the inlet of the chip, bringing the molecules to the region containing the nanowell features. The SEs tubes were synthesized using 400 nM SEs tiles in 1× TAE with 12.5 mM magnesium buffer by thermal anneal. We used a miniPCR machine (mini8X) to hold the tile mixtures at 90°C for 5 min and then performed a series of temperature ramps (−0.5°C/min to 75°C in 30 min, −0.1°C/min to 60°C in 150 min, −1°C/min to 45°C in 15 min, and 0.1°C/min to 40°C in 50 min) with a hold at 40°C for 5 min, followed by a ramp of −0.1°C/min to 31°C in 90 min and a hold at 31°C overnight. The annealed tubes were then diluted and ligated overnight to a final 40-nM concentration.

### Data analysis

The experimental videos were preprocessed using Fiji ImageJ macros for region of interest selection, noise subtraction, and frame averaging. The postprocessing was performed in Python, through a customized data analysis pipeline for CM identification, particle tracking, plotting, fitting, and processing. The mean square displacement and contour identification measurements are described in more detail in section S2.
