# Supplementary material for: Single-molecule capture, release, and dynamical manipulation via reversible electrokinetic confinement (RECON)
Source: Sci Adv. 2025 Sep 17;11(38):eadv8863. doi: 10.1126/sciadv.adv8863 (PMC13155539; doi:10.1126/sciadv.adv8863)
Supplement: Supplementary file 1 — Supplementary Text Figs. S1 to S7 Legends for movies S1 to S6 [file sciadv.adv8863_sm.pdf]

Science Advances
AAAS

Supplementary Materials for

Single-molecule capture, release, and dynamical manipulation via reversible electrokinetic confinement (RECON)

Matheus A. S. Pessôa *et al.*

Corresponding author: Matheus A. S. Pessôa, matheus.pessoa@mail.mcgill.ca

Sci. Adv. **11**, eadv8863 (2025)
DOI: 10.1126/sciadv.adv8863

The PDF file includes:

Supplementary Text
Figs. S1 to S7
Legends for movies S1 to S6

Other Supplementary Material for this manuscript includes the following:

Movies S1 to S6

Supplementary Material

1. Experimental setup

The experimental setup consists of a device containing the field-confinement structures with microfluidic flow-cell, pre-prepared nano analyte containing solution, a 3-D designed and printed resin microscope mount, an indium tin oxide (ITO) coated lid, a waveform generator, and the microscope based imaging platform. The complete experimental setup is shown in Fig. S1. Buffer solution containing fluorescently labeled nano-analytes is loaded into the reservoir inlet and cycled through the device using a manual syringe. The ITO coated lid and device are connected to a function generator via silver wire (50 μm in diameter); the wires are glued to the surfaces using electrically conductive epoxy. Imaging was performed using an inverted microscope (Nikon TiE) with an oil-immersion objective (Nikon CFI Apo 100× NA 1.4) and a Teledyme Photometrics 95B camera. The movie acquisition durations are up to 30 s for each experimental trial, with exposure times that range from 10 ms to 50 ms. The 3-D printed microscope mount was designed using Fusion 360 software and was adapted to the size of the chips, the ITO lid, and the electrical connections. The chips with conducting pads are attached to an ITO-coated lid using a micro-patterned double-sided tape, fixing the distance at approximately 30 μm between the lid and the device (corresponding to tape thickness) with the conductive side of the lid facing the features on the chip. A non-threaded washer is placed on top of the non-conductive part of the lid, and screwed on the chuck to keep the chip and the lid in place while the experiment is conducted. The chip and the lid are then put into contact with the waveform generator (Agilent 33220A) using the silver wire, and the chuck is mounted on the microscope platform.

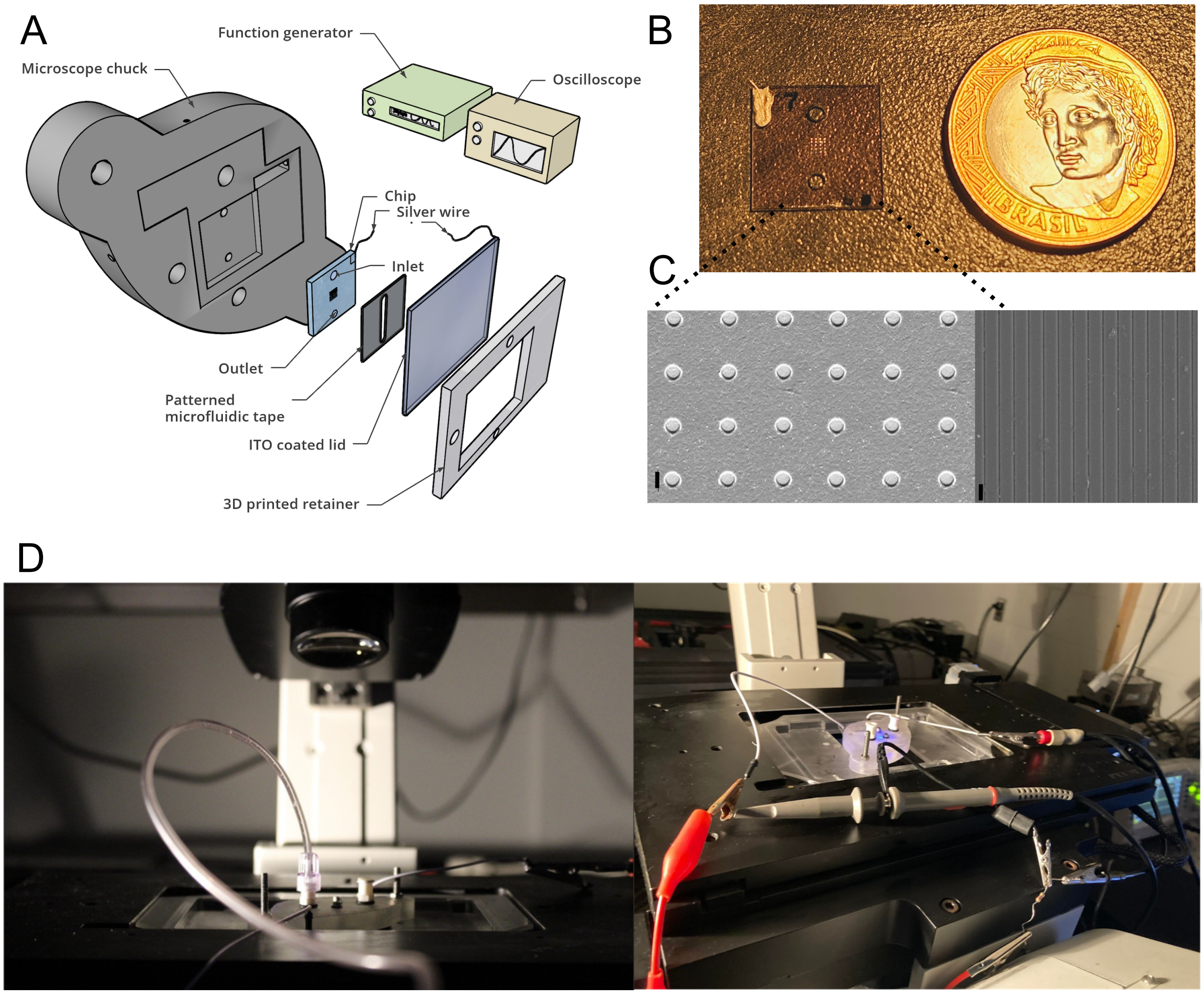

Figure S1: **Experimental setup.** A) A schematic of the chuck for mounting the electrokinetic confinement devices on our inverted microscope platform, showing the device, patterned microfluidic tape constituting the flow cell, ITO coated lid and 3D printed retaining ring. B) The device with flow-cell compared to a one real (R$) coin. C) SEM micrographs showing the nanocavity wells and nanogrooves (scale-bar corresponds to 800 nm). D) The experimental setup showing the chuck mounted on the microscope with pressure and electrical connections.

2. Center of mass fluctuations

We find the fluorescent center of mass (FCM) for each frame using the python package tifffile after applying the same noise subtraction method as presented in Capaldi *et al* and Liu *et al*[7, 33]. The calculation of FCM follows

$$x_{\mathrm{FCM}} = \frac{\sum_x \sum_y x \cdot I(x,y)}{\sum_x \sum_y I(x,y)}, \quad y_{\mathrm{FCM}} = \frac{\sum_x \sum_y y \cdot I(x,y)}{\sum_x \sum_y I(x,y)}, \tag{6}$$

for all pixels in x and y. The overlaid image in Fig. S2 shows the result. We make a histogram of x_{FCM} and y_{FCM}, as shown in Fig. S3, and define the center of the cavity (x_c, y_c). With the center defined, we proceed to find the fluctuations around the cavity center. First, we find the FCM for each frame and compute the fluctuation δr as,

$$\delta r = \sqrt{(x_{\mathrm{FCM}} - x_c)^2 + (y_{\mathrm{FCM}} - y_c)^2}. \tag{7}$$

We then generate the histograms of δr which are then normalized so that the area is equal to 1 along with $\langle(\delta r)^2\rangle$ for each molecule measured. Reported values of $\langle(\delta r)^2\rangle$ correspond to an average of the quantity computed over multiple molecules measured in the array, with an error bar determined by standard-deviation of the mean over the multiple molecule ensemble.

3. Details on tension calculation

When the molecule is partitioned between two cavities, a fluctuating contour linker is formed between them (Fig. 7A). For each frame, we divide the region between the two cavities along the linker (x) by considering one pixel long (110 nm wide) windows along the cavity separation axis (Fig. 7B). We then identify the instantaneous y-excursion of the linker for a given x by taking a transverse cross-section of intensity at the given window and obtaining the y position of the highest intensity along the cross-section. We then obtain the variance of transverse chain excursions (σ_y^2) from the linker y-positions measured at that given x-position across the entire micrograph time-series (Fig. 7B), considering approximately 150 frames for each molecule analyzed.

Inside the cavities we observe small fluctuations in variance that correspond to fluctuations of the molecule within the well. At the cavity edge, there are some fluctuations that correspond to the electric field suppressing fluctuations on the linker that extends outside of the field funnel region. At

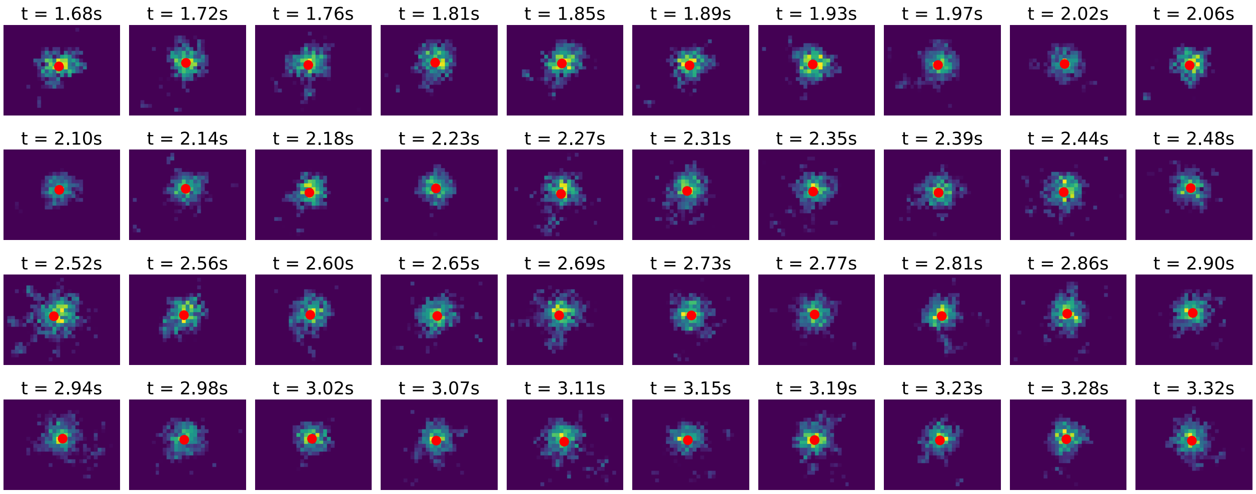

Figure S2: **Tracking of fluorescence center of mass.**Fluorescence center of mass (FCM) overlaid on fluorescent microscopy images of λ-DNA, for electrokinetic confinement at high frequency ($f = 100\,\mathrm{kHz}$) with $V_{\mathrm{max}} = 5\,\mathrm{V}$ and a cavity diameter of 800 nm.

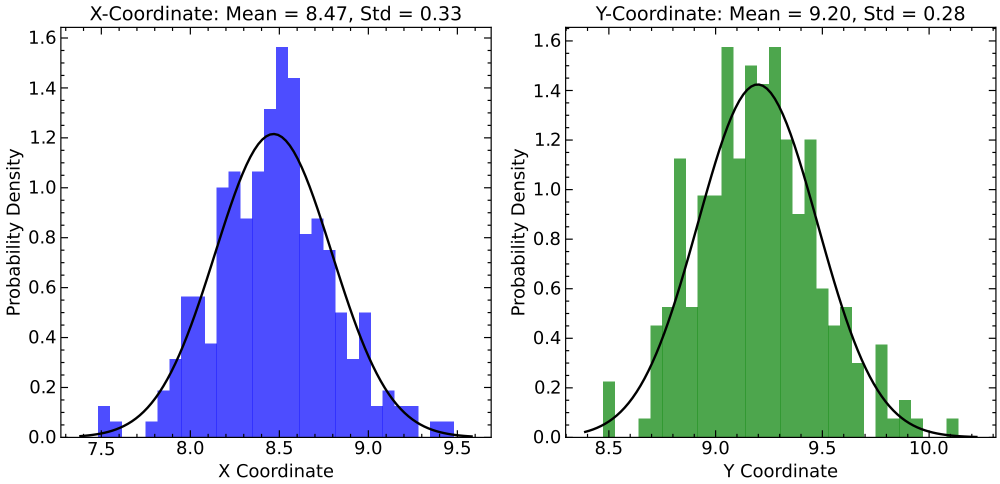

Figure S3: **Distribution of positions to find the center of the cavity.**Defining the cavity well center based on the histogram of FCM positions for x and y.

the center between the two cavities we observe large fluctuations that correspond to free transverse excursions of a chain under uniform tension. These can be fitted by a parabola to find the highest variance value $\sigma_{y,max}$ for each molecule trapped between two wells, for each voltage, with $N = 17$ molecules analyzed in total. Using the tension-fluctuation relation derived in Baba *et al* [56], we compute the force exerted on the DNA based on the fluctuations on $\sigma_{y,max}$.

4. Polymer Partitioning in Electrokinetic Well via Intensity Analysis

An alternative approach to quantifying the degree of confinement of DNA in our device is via the spillover effect, that is how much DNA falls within the strict geometric cavity boundaries and how much spills over as the molecule extends into the larger electric field funnel. First, we define two regions with respect to the molecule FCM: (1) an 'inner' region corresponding to the true cavity area in $x - y$ (with radius equal to the cavity radius) and (2) an 'outer' region that represents the extent of the molecule in $x - y$ beyond the cavity edge (Fig. S4A,B). To find the boundary of the outer region, we find the radial position with respect to the cavity center where the average intensity drops to 20% of the peak intensity (i.e. a natural measure for how far the molecule extends from the cavity center, Fig. S4. The outer region is then defined as the region between the cavity edge and the molecule extent. We can compute the fraction of intensity that falls in the outer region ($I_{\text{outer}}/I_{\text{total}}$, $I_{\text{total}} = I_{\text{inner}} + I_{\text{outer}}$). This measures the portion of polymer that falls outside the cavity boundary (corresponding to polymer occupying regions of the field funnel extending in $x - y$ beyond the cavity edge). We find that $I_{\text{outer}}/I_{\text{total}}$ decreases monotonically as the cavity width increases (Fig. S4d), consistent with our picture that smaller cavities should lead to greater fluctuation of the polymer out of the cavity leading to increased $\langle(\delta r)^2\rangle$.

In Fig. S4e, we present the voltage dependence of $I_{\text{outer}}/I_{\text{total}}$. We observe a general decrease in the outer intensity fraction as the applied voltage increases, a result of the enhanced electric field force that increases the confinement, leading to a greater portion of the DNA molecule remaining inside the cavity. In contrast, higher voltages create deeper potential wells that suppress these fluctuations, leading to tighter confinement and reduced intensity in the region surrounding the cavities.

In Figure S4f, we present the frequency dependence of $I_{\text{outer}}/I_{\text{total}}$. This ratio serves as a proxy for how well the DNA remains confined within the trap at different driving frequencies. At low frequencies, the molecule has time to respond to the field oscillations, allowing partial escape from the cavity. At higher frequencies, however, the field is oscillating too rapidly for the molecule to respond, and consequently the molecule experiences the time-average field, resulting in enhanced confinement. Note that for the smallest cavity diameter (500 nm), the intensity ratio is consistently high across all frequencies, suggesting that the DNA molecule is not fully confined and frequently

spills over beyond the cavity boundary. This behavior reflects the interplay between molecular diffusivity, cavity geometry, and the characteristic time scales of the applied electric field.

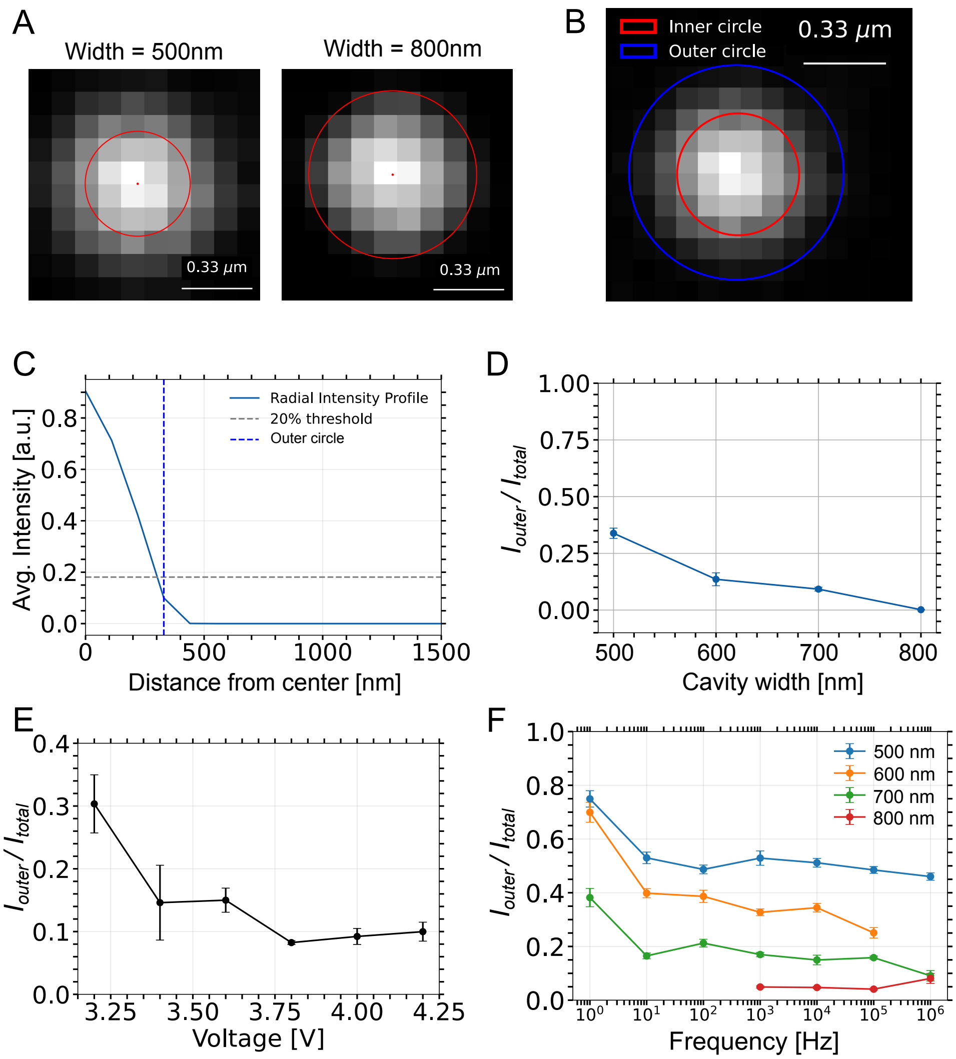

Figure S4: **Quantifying polymer partitioning via fluorescence intensity analysis**. A) Fluorescence micrograph of λ-DNA trapped in nanocavities with $r_c = 250\,\text{nm}$ and $r_c = 400\,\text{nm}$. B) Fluorescence micrograph showing definition of inner circle (indicating cavity edge) and outer circle (indicating molecule extent), from which we can compute I_{outer} and I_{inner} by using the weighted average of the intensity. C) Average intensity as a function of the distance from the cavity center showing location of the outer circle radius. The outer circle radius is defined as the first integer index of the pixel past the point where the average intensity falls below 20% of the normalized average cavity peak intensity. D) The measured $I_{\text{inner}}/I_{\text{total}}$ values shown as a function of cavity width; this decreases monotonically. E) The measured $I_{\text{inner}}/I_{\text{total}}$ values shown as a function of voltage. At lower voltages, approximately a third of the molecule spills over past the cavity edge, whereas at higher voltages the molecule is confined almost entirely within the cavity. F) Measured $I_{\text{inner}}/I_{\text{total}}$ values shown as a function of frequency for cavities of various diameters; a frequency-dependent behavior is observed with decreasing spillover at higher frequencies.

5. IV Characteristics of RECON Devices

Current was measured through a RECON device using a Keithley 2400 SourceMeter. For a low-frequency characterization, the SourceMeter was connected in series with the device, and a 1 Hz square pulse was applied while recording the resulting current. The average DC current, extracted as a function of the applied voltage, is shown in Fig. S5(A). To enable comparison across experiments, the measured current was then scaled by the total area of the active electrode surface (i.e. the exposed area of all well structures etched on the chip), providing an estimate of the current density at the bottom surface of a given well. We observe that, around 2–2.5 V, the device exhibits a sharp increase in current, reaching up to $\sim 100\,\mathrm{pA}/\mu\mathrm{m}^2$. This is consistent with water electrolysis, which is thermodynamically allowed at a minimum cell voltage of 1.23 V (sum of half cell potentials for water splitting) and typically emerges at voltages ~ 2 V due to the overpotential required to drive reaction kinetics and reactant transport [64].

We also conducted high-frequency measurements at $f = 100\,\mathrm{kHz}$ as used in the main experimental results. In this setup, the RECON device was placed in series with both the waveform generator and the SourceMeter (operated in current-sensing mode). A voltage sweep was again applied, and DC current values were extracted for each voltage point. The results are presented in Fig. S5(B), showing the time-averaged current response to a 100 kHz fixed polarity sine wave. While the voltage dependence of the current follows a similar trend to the low-frequency case, the magnitudes are substantially smaller—by approximately two orders of magnitude—reaching $3.8\,\mathrm{pA}/\mu\mathrm{m}^2$ at 3.5 V. This suggests that the advantage of working at high frequency is to provide a low but finite current that can produce a sufficiently strong electric field to drive capture at the wells while minimizing gas evolution.

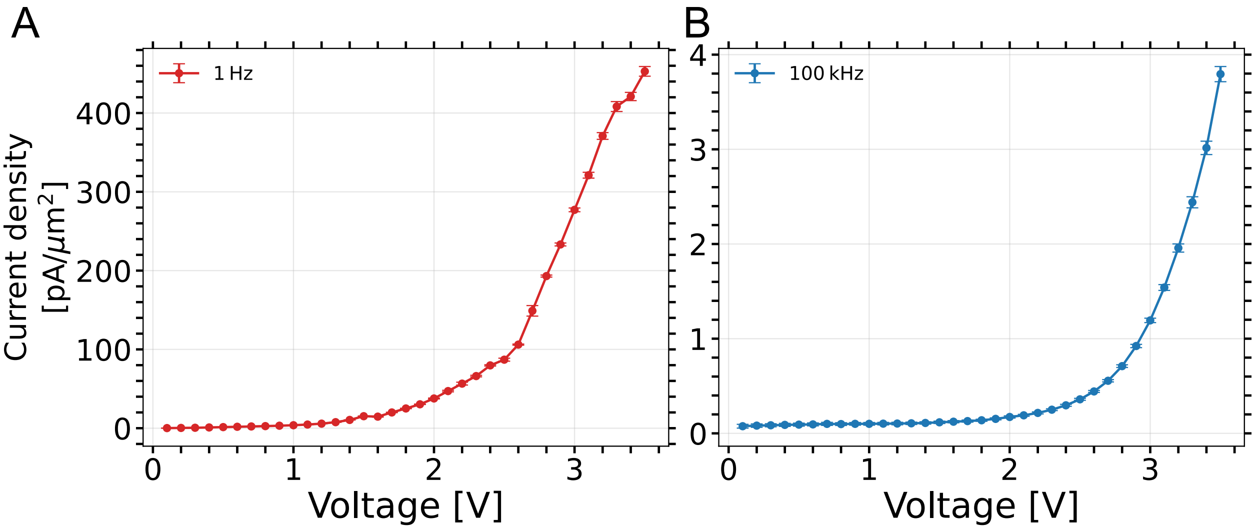

Figure S5: **Current measurements through the RECON device.**Current measurements as a function of applied voltage for a RECON device, (A) at 1 Hz and (B) at 100 kHz. The value of current has been scaled to account for the area of electrode geometries in the nanofluidic chip.

6. Estimation of Electric Field at Electrokinetic Well

We believe that our observations can be qualitatively explained by a simplified picture based on the time-averaged electric field in the well. The presence of a finite time-averaged current flowing through our device implies that there is necessarily a time-averaged current flowing through each well, giving rise to a current density J on the well floor. The current density can be implemented electrostatically via a Neumann boundary condition specifying the electric field at the cavity floor in terms of the local current density $J(r)$ (r radial coordinate) divided by solution conductivity (σ_c). Experimental time scales relating to the trapped nanoentities are ~ 0.1 s (i.e. particle diffusion time across cavity, polymer relaxation time); this time scale is much larger than the $10\,\mu$s signal period at 100 kHz. Thus, from the point of view of the capture and trapping physics, the system necessarily sees the time-averaged electric field.

Here we show how this picture can be used to compute the electric field at the well. In a detailed model the local current density would be determined as a function of the voltage drop across the Stern/compact layer and the ALD layer (ΔV). To build in the electrochemistry this relation could be based on Butler Volmer plus an ALD resistance [72, 73, 74, 75]. Note that $\Delta V = V_0 - V(r, z = 0)$, where V_0 is the voltage applied to the electrode and $V(r, z = 0)$ is the voltage in solution at the cavity floor (i.e. edge of Stern layer). For simplicity, here we use an Ohm's law relation to express current density in terms of voltage drop: $J(r) = [V_0 - V(r, z = 0)]\,/r_{EC}$ where r_{EC} is an areal electrochemical resistance. In a situation with a high to moderate r_{EC}, a large proportion of the voltage will be dropped across the Stern/compact layer, so that maximum voltage in solution at the cavity floor $V_{s,\max}$ is less than the voltage applied to the electrode, i.e. ($V_{s,\max} < V_0$). We introduce a dimensionless potential $V' = V/V_0$ and electric field $\mathbf{E}' \equiv \mathbf{E}/E_0$ where E_0 is a characteristic field $E_0 \equiv V_0/r_c$. The Neumann boundary condition at the cavity floor can then be recast in terms of dimensionless variables as $E'_z = \alpha\,[1 - V'(r, z = 0)]$, with $\alpha = \frac{r_c}{r_{EC}\sigma_c}$ an electrochemical admittance that controls the voltage drop in solution (lower α, lower $V_{s,\max}$, as more voltage is dropped in Stern latyer/ALD layer). We can estimate α from our experimental measurements of current density (Fig. S5(B)). The dimensionless current density $J' \equiv J/(\sigma_c E_0)$. In the limit of low α, $V'(r, z = 0) \ll 1$, so that $J' \approx \alpha$ and α can be estimated via $\alpha = J r_c / V_0 \sigma_c$. Using $r_c = 400\,$nm, $\sigma_c = 0.768\,$mS/cm (measured buffer conductivity) and $J = 3.8\,$pA/μm^2 at

$V_0 = 3.5\,\mathrm{V}$ we find $\alpha \sim 10^{-5}$. Figure S7(A) shows the resulting potential and field lines at the well for $\alpha = 10^{-5}$ (computed via finite element solver FlexPDE in cylindrical coordinates); this is very close to previously reported electric field behavior determined via a fixed voltage boundary condition at the well floor (i.e., $V(r, z = 0) = V_0$ or Dirichlet). The result shown is appropriate for $\alpha = 10^{-5}$ with a cavity with height of 400 nm and width of 800 nm, cavity-to-cavity spacing of $2\,\mu$m and a flow-cell vertical spacing of $30\,\mu$m. This result does not rigorously include the effect of neighboring cavities in the array, which would require modeling the full array geometry in Cartesian coordinates. The effect of the spacing is crudely accounted for here using a full model geometry in cylindrical coordinates consisting of a cylinder equal to the cavity radius plus half the cavity-to-cavity spacing.

Figure S7(B-E) compares in detail the field computed using Dirichlet and Neumann boundary conditions using varying values of the electrochemical admittance. In these plots we normalize $\mathbf{E}'$ to the maximum value of the potential in solution ($V_{s,\mathrm{max}}$) which provides the correct scale of the in-solution potential and field. The Neumann boundary condition gives results very close to Dirichlet boundary conditions for the field behavior far from the cavity and E_z component (Fig. S7(b,); the key difference is that Neumann boundary conditions give a non-zero radial component E_r within the cavity close to the well floor (Fig. S7d, this can drive electroosmosis). Results for low current (overall high resistance/low admittance for electrochemical mechanism) collapse on a master curve when normalized to $V_{s,\mathrm{max}}$ (Fig. S7E).

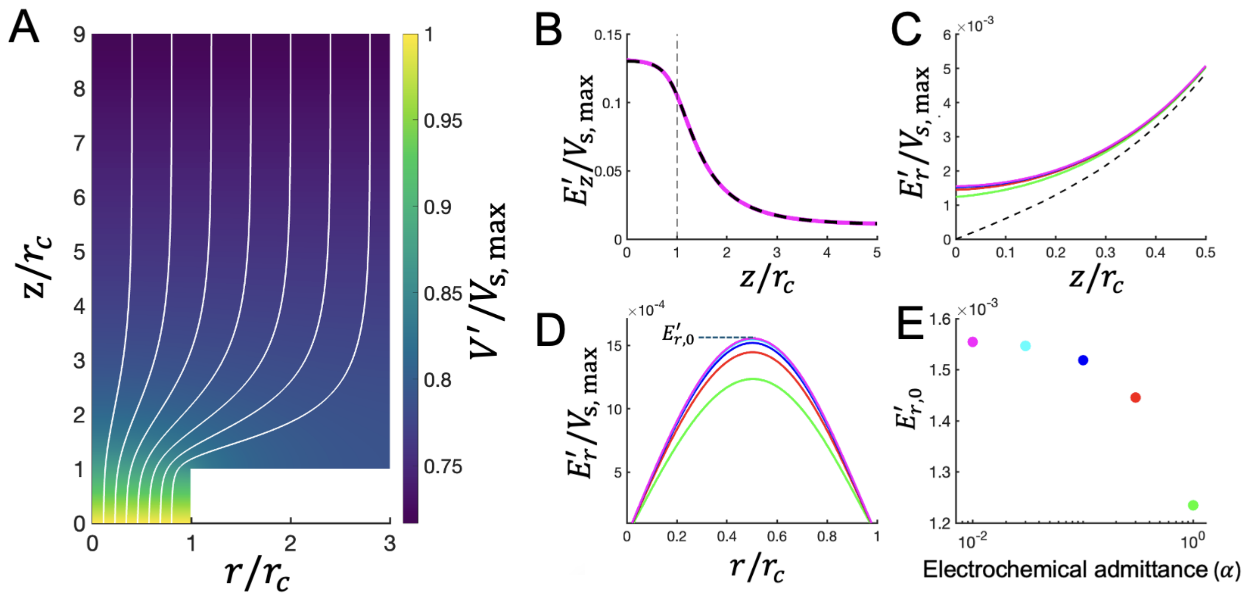

Figure S6: **Estimation of the time-averaged electric field at a cavity**. (A) The electrostatic potential normalized to the maximum potential in solution ($V'/V_{s,\max}$) shown as a color-scale with superimposed electric field-lines indicating the field-funnel structure. The model field is appropriate for $\alpha = 10^{-5}$ with a cavity with height of 400 nm and width of 800 nm and a flow-cell vertical spacing of 30 μm. (B) Dimensionless z-component of electric field E'_z versus z-position normalized to cavity radius r_c. The result for Dirichlet boudary condition ($V(r, z = 0) = V_0$) shown as blacked dashed-line; result for Neumann boundary conditions correspond to colored curves (green, $\alpha = 1$; red, $\alpha = 0.3$; blue, $\alpha = 0.1$; cyan, $\alpha = 0.03$; magenta, $\alpha = 0.01$). All curves overlap in this case. (C) Dimensionless radial component of electric field E'_r at cavity center $r = 0$ versus z-position normalized to cavity radius r_c. (D) Dimensionless radial component of electric field E'_r at cavity floor ($z = 0$) versus radial position normalized to cavity radius r_c. In this case, Dirchlet boundary conditions necessarily give $E'_r = 0$. (E) Max radial field value from (D) $E'_{r,0}$ plotted versus the electrochemical admittance (α). The results for decreasing α collapse onto a single curve.

7. Molecular Dynamics simulations

We performed simulations using `EspressoMD`, version 4.2, to investigate electrokinetic confinement of a polymer chain in a cylindrical well. The DNA is modeled as a charged bead-spring polymer (Fig. S7A). We assume that the vertical (z) component of the electric field in the well is constant everywhere in space and that there is no radial field component (Fig. S7B). The field was also assumed constant in time (i.e. a DC signal was used), which is justified by the large separation in timescales between field modulation (10^{-2}–10^{-6} s) and scale for the polymer center-of-mass (CM) diffusion within the cavity ($\tau_d \sim 0.1$ s), so that the polymer sees the time-averaged field.

The λ-DNA molecule is modeled as a single negatively charged ($q = -1$) bead-spring polymer composed of $N = 70$ monomers, resulting in a radius of gyration $R_g = 5.195$ simulation units. Bonded interactions are enforced via a finite extensible nonlinear elastic (FENE) potential described via,

$$U_{FENE} = -\frac{1}{2}kR_0^2 \ln\left(1 - \frac{r^2}{R_0}\right), \tag{8}$$

with equilibrium bond length $R_0 = 0.3$, spring constant $k = 10.0$, and maximum bond extension $d_{r,\max} = 2.0$. Non-bonded monomer–monomer and monomer–wall interactions are modeled by a truncated Lennard-Jones potential (WCA),

$$U_{WCA} = 4\epsilon\left[\left(\frac{\sigma}{r}\right)^{12} - \left(\frac{\sigma}{r}\right)^{6} + \frac{1}{4}\right], \tag{9}$$

initially set with $\epsilon = 1.0$ and $\sigma = 0.3$, which is gradually increased to $\sigma = 1.0$ over a warm-up phase to prevent numerical instabilities.

Polymer stiffness is introduced halfway through the warm-up phase via a bending potential

$$U_{\text{bend}} = \kappa[1 - \cos(\theta - \theta_0)], \tag{10}$$

with $\phi_0 = \pi$ and bending modulus $\kappa = 0.48$, resulting in an angle-based energy penalty that enforces local chain rigidity (see Fig. S7a for defintion of θ).

The polymer is confined within a cylindrical cavity of fixed height and radius $R_{\text{well}} = 3.0$ simulation units, ensuring a confinement ratio $R_g/R_{\text{well}} = 1.73$ for this cavity size ($R_g = 5.195$ in simulation units). As the physical λ-DNA $R_g = 700$ nm, this cavity size corresponds to a 800 nm diameter cavity ($r_c = 400$ nm). We then vary the cavity diameter in our simulation model

in proportion to the variation in cavity diameter used in experiments. The walls of the pore are impenetrable and interact with the polymer via WCA repulsion. A uniform electric field of strength $E = 2.0$ is applied along the z-axis, acting on each charged monomer. This potential is described by

$$U_{\text{el}} = -qEz, \quad F_{\text{ele}} = qE\hat{z}.$$

The time evolution of each monomer obeys the Langevin equation:

$$m\frac{d^2\mathbf{r}_i}{dt^2} = -\gamma\frac{d\mathbf{r}_i}{dt} + \mathbf{F}_i^{\text{WCA}} + \mathbf{F}_i^{\text{FENE}} + \mathbf{F}_i^{\text{bend}} + \mathbf{F}_i^{\text{el}} + \boldsymbol{\eta}_i(t), \tag{11}$$

where $\mathbf{r}_i$ is the position of monomer i, $m = 1$ is the particle mass, $\gamma = 1.0$ is the friction coefficient, and $\boldsymbol{\eta}_i(t)$ is a stochastic noise term satisfying

$$\langle \eta_i^\alpha(t)\eta_j^\beta(t')\rangle = 2\gamma k_B T\delta_{ij}\delta^{\alpha\beta}\delta(t - t'),$$

with $k_B T = 0.2$ the thermal energy. Simulations were run for 3000 cycles, each consisting of 200 integration steps. At the end of each cycle, the positions of all monomers were recorded, and the polymer center of mass (COM) was computed and stored for post-processing. Each condition was repeated 25 times to generate ensemble statistics and estimate error bars.

Figure S7d shows simulated values for the variance in polymer CM fluctuations about the cavity center ($\langle(\delta r)^2\rangle$) as a function of cavity diameter (Fig. S7d, note that the cavity diameter is scaled to physical units using the R_g). We observe that $\langle(\delta r)^2\rangle$ decreases as the cavity width increases, consistent with our experimental observations.

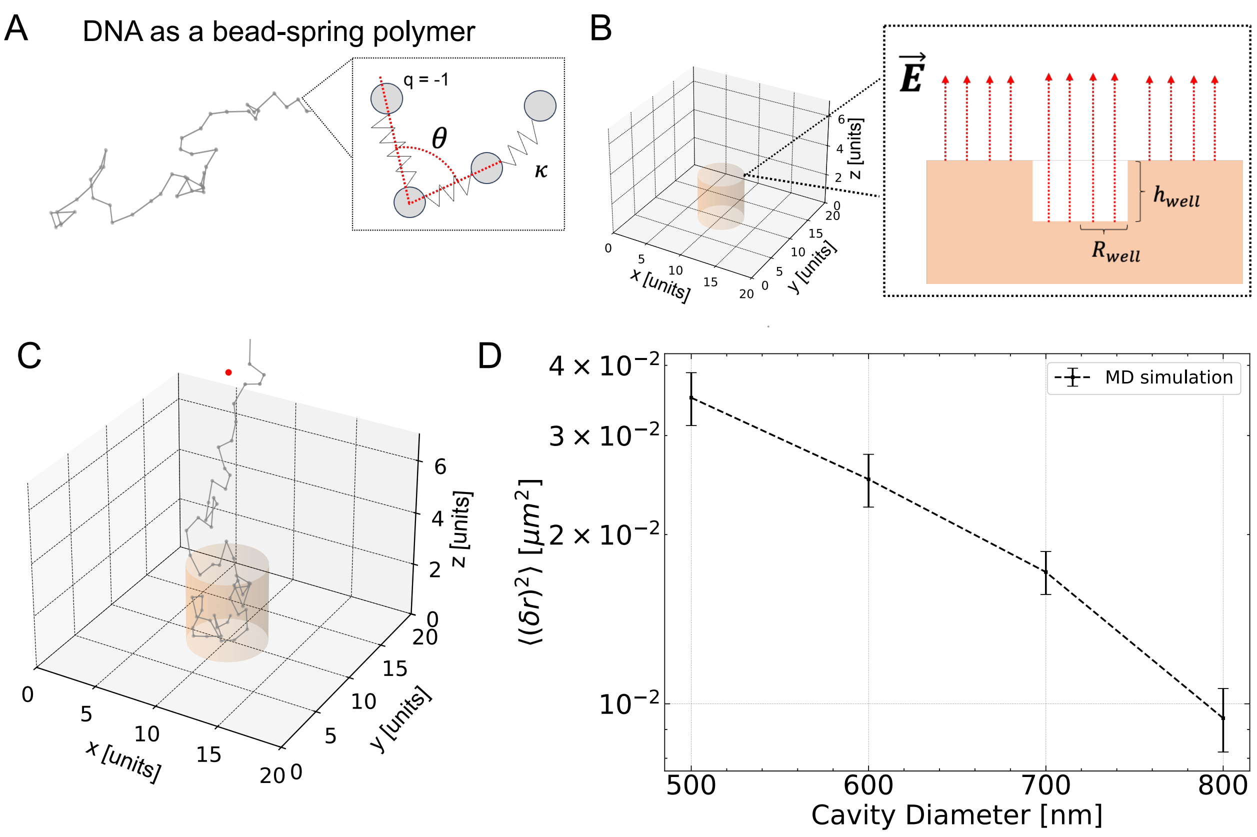

Figure S7: **Molecular dynamics simulations.**(A) The DNA is modeled as a charged bead-spring polymer with bending modulus κ and local chain rigidity. The angle θ is used to define the bending potential U_{bend}. (B) The cylindrical cavity is defined in simulation units, with the electric field pointed in the z direction, thus attracting the negatively charged polymer to the inside of the well. During the simulation run, we track the center of mass (in red) as a function of time steps during capture, shown in C), and during confinement. D) By using the relation between diameter of the cavity relative to the polymer radius of gyration, we convert the cavity diameters to nanometers, and compute the variance of the center of mass for different cavity diameters used in the simulation. The results show agreement with the experimentally obtained trends of decreasing variance for increasing cavity diameter.

Captions for Movies S1-S6

Caption for Movie S1. Reversible confinement of λ-DNA molecules in array of wells. In this experiment, d = 400 nm), with $V_{\max}$ = 5 V, and f = 100 kHz.

Caption for Movie S2. Influence of different frequencies on λ-DNA confinement. Confinement dynamics of λ-DNA molecules at frequencies f = 1 Hz, f = 10 Hz, and f = 100 Hz in wells of radius r_c = 350 nm using an ACfp sine wave ($V_{\max}$ = 5 V).

Caption for Movie S3. λ-DNA confinement under sinusoidal amplitude modulation, at different frequencies. Dynamics of λ-DNA under confinement in wells (r_c = 400 nm) and extracted δr using amplitude modulated (AM) ACfp sinusoidal wave, with $V_{\max}$ = 4.0 and $V_{\min}$ = 2.5 V, at f = 100 kHz, using different f_m (1 Hz, 2 Hz, 3 Hz).

Caption for Movie S4. Confinement and release of T4-DNA molecules. Reversible molecular partitioning of a T4-DNA molecule confined between two wells (r_c = 400 nm), using an ACfp sine wave ($V_{\max}$ = 4 V, and f = 100 kHz).

Caption for Movie S5. Influence of voltage in varying fluctuations of multi-well states of confined T4-DNA. Molecular partitioning of a T4-DNA molecule confined between two wells (r_c = 400 nm), using an an ACfp sinusoidal wave with f = 100 kHz, at voltages $V_{\max}$ = 3.3 V, and V_{max} = 4.2 V.

Caption for Movie S6. Variable confinement of T4-DNA under stochastic driving. Dynamics of a DNA molecule under stochastic driving in a nanowell array (in different colors, each color representing a well), with an ACfp Gaussian noise centered at 2.9V and standard deviation 0.66V. On the bottom right, the total well occupancy as a function of time.
